# Pedestrian POSE estimation using multi-branched deep learning pose net

**DOI:** 10.1371/journal.pone.0312177

**Published:** 2025-01-24

**Authors:** Muhammad Alyas Shahid, Mudassar Raza, Muhammad Sharif, Reem Alshenaifi, Seifedine Kadry

**Affiliations:** 1 Department of Computer Science, COMSATS University Islamabad, Wah Campus, Islamabad, Pakistan; 2 Namal University, Mianwali, Pakistan; 3 Department of Information Technology, College of Computer Sciences and Information Technology, Majmaah University, Majmaah, Saudi Arabia; 4 Department of Computer Science and Mathematics, Lebanese American University, Beirut, Lebanon; 5 Noroff University College, Kristiansand, Norway; Wuhan University of Science and Technology, CHINA

## Abstract

In human activity-recognition scenarios, including head and entire body pose and orientations, recognizing the pose and direction of a pedestrian is considered a complex problem. A person may be traveling in one sideway while focusing his attention on another side. It is occasionally desirable to analyze such orientation estimates using computer-vision tools for automated analysis of pedestrian behavior and intention. This article uses a deep-learning method to demonstrate the pedestrian full-body pose estimation approach. A deep-learning-based pre-trained supervised model multi-branched deep learning pose net (MBDLP-Net) is proposed for estimation and classification. For full-body pose and orientation estimation, three independent datasets, an extensive dataset for body orientation (BDBO), PKU-Reid, and TUD Multiview Pedestrians, are used. Independently, the proposed technique is trained on dataset CIFAR-100 with 100 classes. The proposed approach is meticulously tested using publicly accessible BDBO, PKU-Reid, and TUD datasets. The results show that the mean accuracy for full-body pose estimation with BDBO and PKU-Reid is 0.95%, and with TUD multiview pedestrians is 0.97%. The performance results show that the proposed technique efficiently distinguishes full-body poses and orientations in various configurations. The efficacy of the provided approach is compared with existing pretrained, robust, and state-of-the-art methodologies, providing a comprehensive understanding of its advantages.

## 1. Introduction

Vision-based pedestrian recognition [1–3] is applied widely, including video surveillance [4,5], driving assistance [6,7], human-machine interaction [8], advanced robotics, video indexing [9], and automated driving systems [10]. Recognizing a pedestrian has always been critical to achieving an accurate and precise safety system [11]. Human pose estimation (HPE) [12] is perhaps the most challenging field and a hot topic of study for researchers [13]. An image or video of a human being estimates the posture, orientation, position, or three-dimensional (3D) [14] human body placement. 2D or 3D imaging techniques [15] are used in HPE to determine the pose, orientation, action, location of human body parts[16], and facial expression [17]. The prime and primary goal of HPE is to recognize a human body and its parts, i.e., the head, hand, elbow, arm, wrist, ankles, and shoulders. These parts are crucial/critical for analyzing the human in images. In graphic applications, e.g., animated movies, action recognition, biometrics [18,19], simulations, and games, human positions and orientations are needed to estimate. In such graphics-based applications, HPE is particularly critical to analyze. A bunch of cameras is required in some existing systems. These are used for HPE to cover the identified area. Sometimes, stationary camera applications are also used, which are expensive. With that, some calibrated and traditional cameras can also be used for motion-based recognition [20].

**Fig 1:**
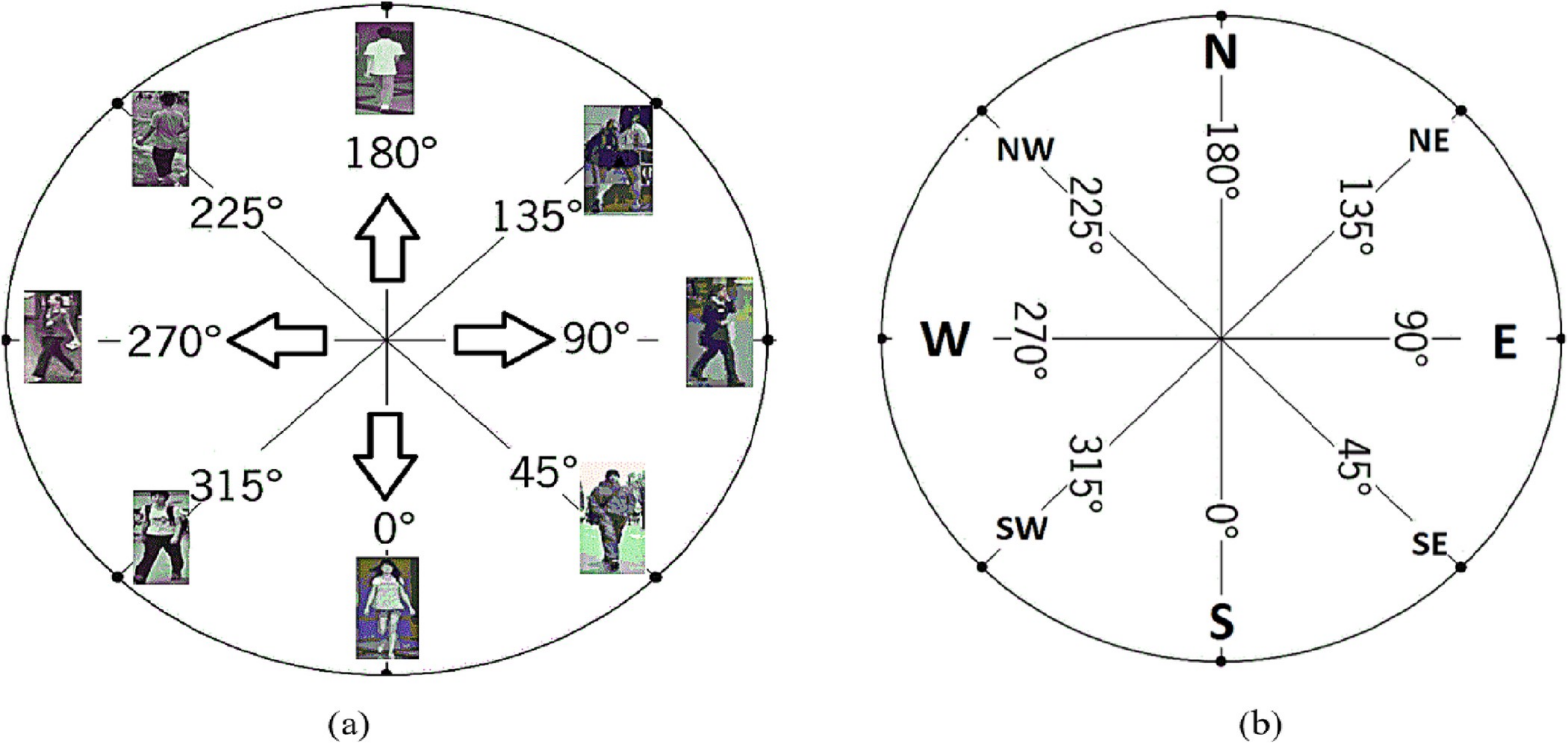
(a) People’s perspectives with different orientations and pose angles (b) 0° South (Front), 45° South East (Right Front), 90° East (Right), 135° North East (Right Back), 180° North (Back), 225° North West (Left Back), 270° West (Left), 315° South West (Left Front) (the pedestrian images in this diagram are taken from PKU-reid [42], BDBO [43], and TUD [44,45] datasets).

One of the most significant, primary, essential, and critical issues is the complexity of viewpoint variation. For computer vision researchers, HPE has always remained a challenging task due to variations and dissimilarities in appearance, with occlusion, hardly visible joints, and minor joint problems [21]. Therefore, different camera angles are used in different scenarios [22]. HPE is being used in many real-world applications like pedestrian orientation and action recognition [23], analysis of medical images [24], human-computer interaction[25], understanding of human behavior [26], surveillance, sports, and biomechanics [27]. In the intelligent video surveillance system, deep learning technologies are applied for human activity recognition (HAR) [28–31]. It predicts human behaviors and estimates human poses [32]. To estimate the pose of a human or pedestrian, its orientation is vitally essential in computer vision research. The most crucial task in the human body orientation estimation (HBOE) [33] challenge is to accurately evaluate the direction of motion of the pedestrian in a provided video or photograph. Accurate HBOE can significantly improve the estimation of human posture as a vital component of the behavior analysis system [34]. Estimating the human body’s directions or a specific part of it is significant for a variety of activities, for example, healthcare scenarios [35], counting people [36], detecting a fall [37], remotely tracking a patient’s recovery [38], predicting a fall in an older adult [39], robot-human interaction because robots work and orient themselves to watch and interact with humans more naturally [40]. Security cameras can more precisely identify people’s behavior. The body’s position, by indicating the walking directions of the pedestrian, is an excellent approach to predicting what the pedestrian is likely to do next in autonomous driving [41]. Fig 1**(A)** depicts people’s perspectives from several cameras with different orientations and pose angles. Fig 1**(B)** depicts pose angles from several cameras with different orientations like 0° South (Front), 45° South East (Right Front), 90° East (Right), 135° North East (Right Back), 180° North (Back), 225° North West (Left Back), 270° West (Left), 315° South West (Left Front).

In computer vision, HBOE is the most critical issue investigated primarily in pedestrian safety and activity prediction [46] and robotic applications [47]. The direction of interest in a video surveillance system is dictated by the orientation of the human body or head [48]. For effective social engagement, body and head alignment and movement are essential nonverbal communication skills. Some individuals with social communication difficulties may struggle to display normative nonverbal communication indicators, such as maintaining regular eye contact and consistently orienting their body towards a speaker. [49]. Red, green, and blue (RGB) [50] cameras are used in most scenarios of body orientation estimation due to their low cost [51]. However, RGB-D cameras, such as Intel RealSense [52] and Microsoft Kinect, are also used. When there isn’t enough data to extract relevant features, features are constructed and work admirably [52]. Aside from the handcrafted components, in any end-to-end learning system, an automatic feature extraction technique can also address most classification tasks’ issues. At the same time, a deep CNN-based model has been verified to be more competitive in object recognition tasks [53]. To determine the human body orientation [54–57], numerous systems used for tracking motion have been established, including the LIDAR system [60] used as motion trackers [61] and some mechanical [62] and optical motion trackers [58]. HBOE utilizing RGB photographs has proved to be of significant development in current years and has been successfully performed in a limited number of cases [59]. However, uncontrolled pose obstacles for RGB-based systems, such as illumination alterations, occlusions, and a significant change in posture, have reduced their effectiveness.

CNN-based techniques are used to resolve a variety of performance difficulties. Therefore, a deep learning CNN-based method is created in this proposed approach to estimate human pose appearances. The following are the primary contributions of this manuscript:

- As input, grayscale images are used for the proposed model, which is taken with 2D cameras. Because there is some noise in standard images, the results are compromised. Dehazing is used as preprocessing to clear the hazy, low visibility/low-resolution/degraded/low visible images to improve the visibility of the images.
- A proposed CNN-based system comprising 66 layers with B1, B2, and B3 branches for feature extraction to depict appearance-based orientation and full-body pose estimation. Both still images and image sequences are used in the proposed system.
- Ant colony selection (ACS) [60] is used to select an optimized feature set that set is provided to nine (9) different classifiers to classify and estimate the full-body pose of a pedestrian. The outcomes are assessed using existing techniques, and promising results are obtained.

The leading organization of the manuscript is structured as section 1 is the introduction. This section provides an overview of the problem being addressed, the motivation behind the research, and the objectives of the study. It sets the stage for the rest of the manuscript by outlining the significance and scope of the work. The section 2 is related work. It highlights the key findings, methodologies, and gaps in the current knowledge that the present study aims to address. By doing so, it establishes the context and foundation for the research. Section 3 details the methods and materials used in the study. This includes a comprehensive description of the proposed CNN framework, the architectural setup, and the implementation specifics. It provides the necessary technical details that allow for replication and validation of the work. The section 4 is proposed datasets. This section introduces the newly created large dataset for full-body pose and orientation classification. It discusses the dataset’s composition, the data collection process, and the features that make it suitable for training and evaluating the proposed CNN technique. Section 5 discusses experiments and results. The manuscript presents the experiments conducted. It includes the experimental setup, the metrics used for evaluation, and a detailed analysis of the results obtained. This section demonstrates the effectiveness and robustness of the proposed method through quantitative and qualitative assessments. The final section, 6, concludes the manuscript.

## 2. Related work

The highest-semantic feature embedding [61] focuses on existing techniques, ignoring the insights concealed in prior layers. Furthermore, due to misalignment and stance fluctuations, pose-related information must be utilized entirely. [62]. Numerous studies on human pose estimation have been conducted. There are two sets of HPE approaches such as (a) top-down [63] and (b) bottom-up [64]. Top-down [65] and bottom-up approaches [66] are employed in the provided work to discover all essential points before grouping them into distinct instances. According to the estimated people count in the image, HPE is assigned to a group of people or a single person.

Compared to multi-person and multiview pose estimation, estimating the pose of a single person from a given image that may include more than one person is substantially more accessible (and usually does) [67]. Both approaches can also determine an individual’s posture and pose in an image. For single-person channels, deep learning techniques can also be divided into regression-based approaches [68] and body part detection-based approaches [68]. Regression models develop a mapping from an input image to body joints or model parameters using an end-to-end approach [69]. Body part detection algorithms aim to estimate the relative positions of various joints and body parts [70], commonly managed by heatmap representations [71]. Research relies on regression systems, such as [72], which proposed DeepPose [73], a cascaded deep neural network regressor, to estimate joints and body parts from photos. As a result of DeepPose’s exceptional performance, the HPE research framework shifted away from traditional methodologies and toward deep learning, notably CNN [74]. Based on GoogleNet [75], an Iterative Error Feedback (IEF) [76] network is presented that inoculates estimation errors back into the input space to modify an initial response gradually.

For 2D human poses, the top-down technique perceives the number and position of a person and then finds each individual. Top-down approaches receive benefits from the advantages of bottom-up approaches. Several well-known strategies for estimating the pose of a single person are already present. Still, they accept early obligations: if the single-person detector fails, they will accept it first. As a frequent case when individuals are in close quarters, there is no prospect for a return. In the early years, to predict the 3D poses of humans, a set of handcrafted features, geometric features, and perspective relationships were employed. In recent years, the use of deep neural networks for images to 3D HPE has expanded mainly due to considerable developments in deep learning methodologies [77]. Estimating the human body or its shape from color photos is difficult. A unique work is presented in [78] to analyze the shape of the human body from numerous color images using off-the-shelf segmentation [79] without regard for stance, backdrop, or camera viewpoint. From a single RGB image, to determine the 3D human pose, a framework is presented in [80]. The reconstruction network, depth map, 2D pose estimator, and monocular image establish a dynamic and user-friendly system. In [81], metric-scale truncation-hearty (MeTRo) pose estimation and volumetric heatmaps for root-relative are presented. Instead, it is also being used to restrict the image space. A complete convolutional neural network is also expected to address the space measurement of a person’s location directly.

Two main approaches to estimating 3D human pose [82] for a single person are known as model-free and model-based [82] approaches. Whether or not they estimate 3D human position using a human body model [83]. 3D multi-person HPE comprises Top-Down approaches that use a human detection network to identify single-person zones. The region can be calculated for individual 3D poses for every single person using a 3D pose architecture. The global coordinate is then utilized to align the three-dimensional postures. Firstly, bottom-up approaches estimate all joints and depth maps of those joints. Those body parts are related to each individual part’s relative depth and root depth [84].

The existing techniques used for HPE are pointing out some differences and limitations. A few techniques neglect key insights from earlier layers that contain crucial pose-related data. Some of them do not extensively address misalignment and stance fluctuations information, which may lead to mistakes in pose estimation. In crowded environments, in particular, top-down techniques may not work if the initial single-person detector does not work. Estimating 3D stances from color photographs is challenging since there is little depth of information. Many techniques start with the assumption that there is just one person present. A mistake in this assumption could result in serious mistakes. Accurate reconstruction of 3D poses depends on depth information, which is absent when estimating human stances from 2D photos. The complexity of multi-person pose estimation arises from the necessity to accurately estimate the poses of numerous individuals while maintaining individual distinction. People nearby might cause posture estimate mistakes because body part overlapping is difficult for algorithms to distinguish. Even with the advances, deep learning models today are still struggling to anticipate poses correctly in complicated settings like changing camera angles and occlusions.

The proposed method incorporates advanced and innovative strategies that enhance performance, addressing several limitations of existing pedestrian full-body pose and orientation estimation techniques. Typically applied to color images, traditional models often struggle with noise and feature selection. In contrast, the proposed approach utilizes grayscale images and includes dehazing as a preprocessing step, significantly improving accuracy and visibility. The method features a 66-layer CNN model with three distinct branches (B1, B2, and B3), which excels at capturing complex features such as poses and orientations and effectively handles still images with diverse backgrounds.

Additionally, feature optimization using ACS ensures that only the most relevant and appropriate features are utilized. This has enhanced the robustness, accuracy, and efficiency of classification. Cross-validation was conducted on three distinct datasets, where the proposed method outperformed the state-of-the-art models. The method’s average 95% and 97% accuracy across various configurations demonstrate its effectiveness, robustness, and efficiency.

## 3. Proposed methodology

**Fig 2:**
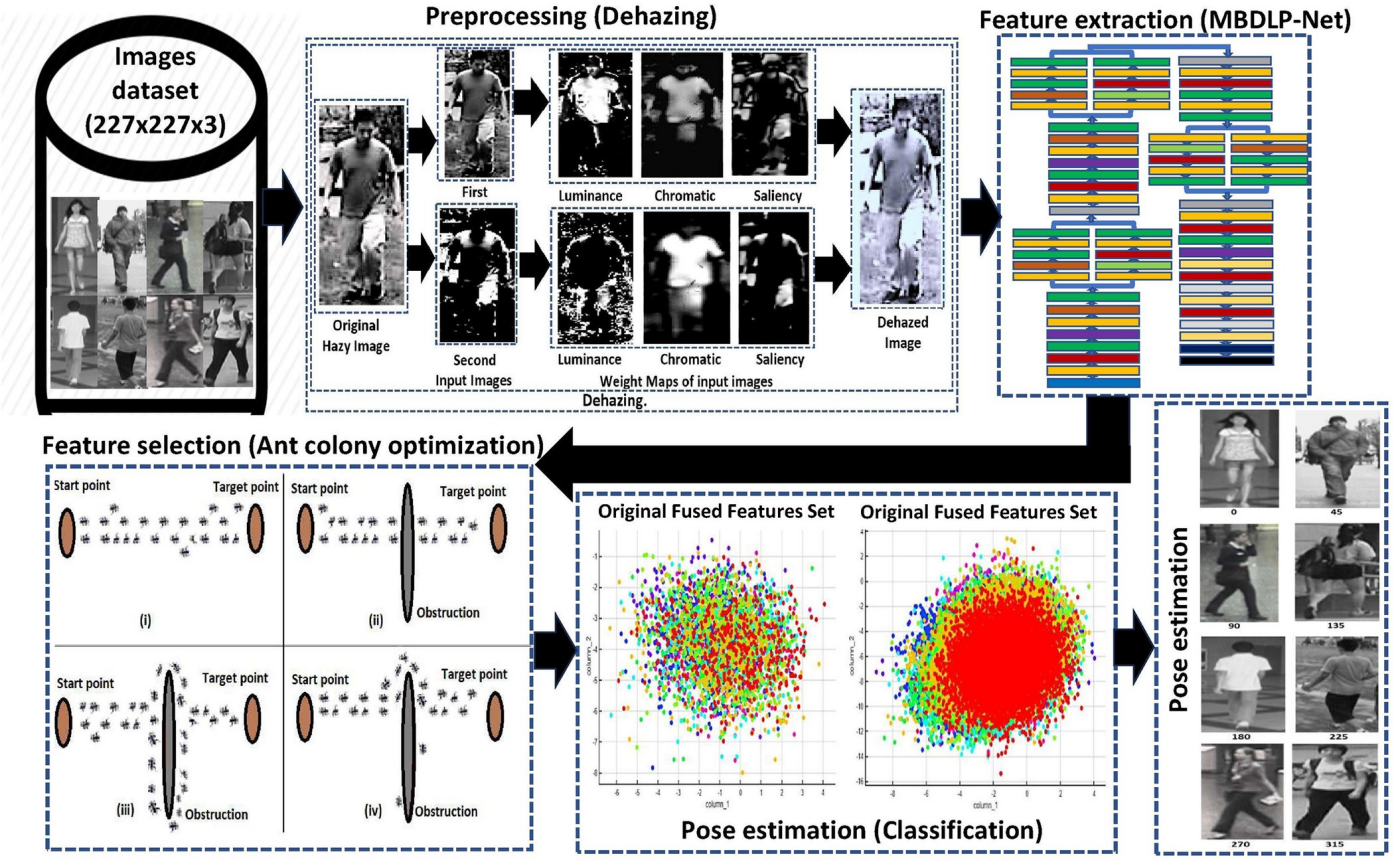
Provide a complete overview and representation of the proposed paradigm used for pose estimation. (The pedestrian images in this diagram are taken from BDBO, PKU-reid, and TUD datasets).

This portion provides a comprehensive outline of the proposed CNN architecture. Fig 2 depicts a complete sketch of the proposed approach paradigm.

The description of the proposed 66-layer CNN model is also included in this section. The primary processes of the proposed CNN framework, including pre-training with the dataset CIFAR-100 [85] with 100 different classes, are also included. Preprocessing (dehazing), feature extraction from the extensive dataset for body-orientation (BDBO) [43], PKU-Reid [42], and TUD multiview pedestrians [44,45] datasets by using proposed deep learning-based CNN model, selection of feature subsets using the ant colony selection, and classification/estimation by using different classifiers is also elaborated in this section.

### 3.1 Preprocessing

Image pre-processing is crucial for preparing images in deep learning for tasks like training, testing, and inference. It involves resizing, orientation, color corrections, and haze removal. Dehazing is employed as a preprocessing step to reduce haze in images, resulting in improved visibility and smoother appearance. Haze removal is challenging but essential for computer vision and photography in low-light or bad-weather scenarios. Researchers have focused on obtaining high-quality dehazed images in the past decade. Poor weather conditions, such as haze, fog, mist, or smog, reduce color and contrast in images. In computer vision, Eq (1) is usually used to describe the formation of a foggy or hazy image [85].


I(x)=J(x)t(x)+A(1−t(x)) (1)

**Fig 3:**
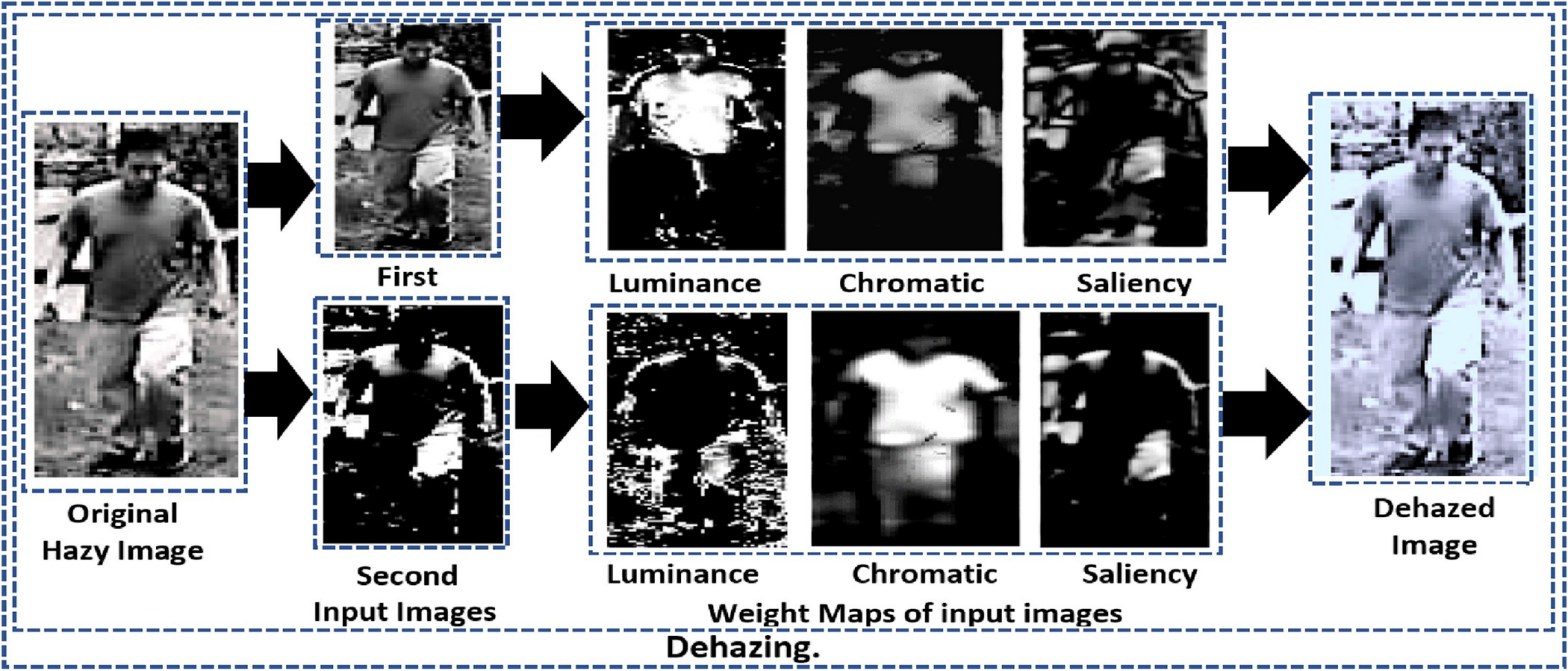
Complete illustration of the dehazing process.

In Eq (1), the hazy image is represented by I(x). In contrast, J(x) represents the reconstructed dehazed image, A represents the air lightly, and the transmission is represented by t(x). The transmission t(x) is the amount of light that does not scatter and reach the camera. The amount of light that survives and makes it to the camera is also essential. The basic steps in the dehazing [86] process are as follows: Hazy images are processed to generate image inputs and weighted maps. The weight maps from the first input are used as subsequent weight maps for the second input. Normalized weight maps are created from these resultant weight maps. Gaussian and Laplacian pyramids are applied to the normalized weight maps of derived input images. The outcome is an improved dehazed image suitable for utilization in deep-learning models for further image processing. Fig 3. depicts the complete illustration of the dehazing process. The dehazing process inversed the model as follows to restructure the dehazed image as shown in Eq (2):

$$J(x)=\frac{I(x)-A}{t(x)}+A$$ (2)

During the dehazing process, three image maps are generated: luminance, chromatic, and saliency weight maps. The luminance weight map assigns higher values to brighter regions and lower values to darker regions, aiding in contrast enhancement, brightness adjustment, and visibility improvement in image dehazing. One standard method for generating a luminance weight map is based on the local contrast and frequency content of the image, which can be expressed using the following equation:

L(x,y)=(R(x,y)+G(x,y)+B(x,y))/3 (3)

In Eq (3), at pixel (x,y), L(x,y) is the luminance value, and R(x,y), G(x,y), and B(x,y) are the values of red, green, and blue colors at the same pixel. The range and sensitivity of the weight map control the constants k and σ.

The chromatic weight map is derived from the color content of an image, assigning higher values to regions with rich or saturated colors expressed as.

C(x,y)=max(R(x,y),G(x,y),B(x,y))−min(R(x,y),G(x,y),B(x,y)) (4)

Where C(x,y) is the chromatic value (x,y). R(x,y), G(x,y), and B(x,y) are RGB colors. Attention-grabbing features are captured by the saliency weight map in an image by analyzing visual cues like color contrast, texture, and object boundaries. Higher values are assigned to image dehazing, which signifies the haziness in the image and is applied to restore colors and contrast. A standard method for generating the saliency weight map in image dehazing is based on contrast and color statistics, as expressed in a specific equation.


S(x,y)=exp(−k*[max(R(x,y),G(x,y),B(x,y))−min(R(x,y),G(x,y),B(x,y))]/σ) (5)

S(x,y) is the saliency value, and k and σ are constants controlling the saliency range and sensitivity.

### 3.2 Acquisition of deep features

The proposed framework will extract features using a deep learning-based trained CNN framework. For this purpose, a third-party pre-existing dataset, such as CIFAR-100, is used for pre-training. CIFAR-100 is an image dataset containing 100 different classes. For learning purposes, 500 images are used in each class. For validation, 100 images are employed in each class. The images used for training and validation are also intermixed and used in pre-training. Therefore, in each class, 600 images are presented for training.

#### 3.2.1 Proposed MBDLP-Net

**Fig 4:**
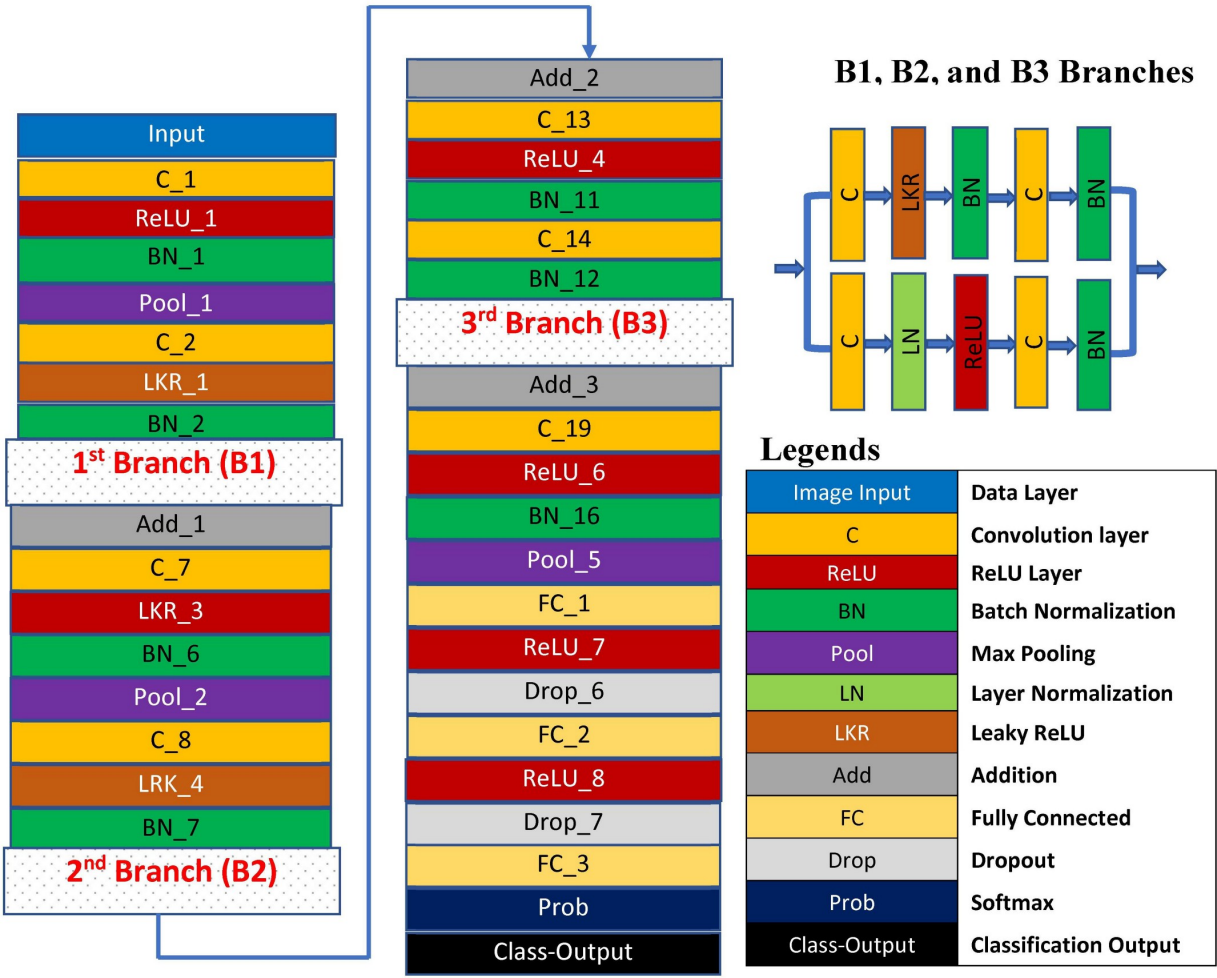
Graphical representation of the proposed MBDLP-Net.

A CNN-based model with 66 layers is proposed. Multi-branched deep learning poses net (MBDLP-Net) refers to the whole architecture of the presented deep CNN network. Fig 4 represents the graphical representation of the proposed MBDLP-Net. Table 1. depicts the arrangement and entire formation of the layers of the proposed MBDLP-Net.

**Table 1:** Arrangement and entire formation of the layers of the proposed MBDLP-Net.

L#	Layer	Name	Activations	Learnable
1	Data 227x227x3 image	Image Input	227*227*3	-
2	C_1, 96 11x11x3 Conv,strd [4 4), pdng [0 0 0 0)	Conv	55*55*96	Wts 11*11*3*96, Bias 1*1*96
3	Relu_1	ReLU	55*55*96	-
4	BN_1, 96 Chnl	Batch Norm	55*55*96	Offset 1*1*96, Scale 1*1*96
5	Poon 3x3, strd [2 2), pdng [0 0 0 0]	Max Pooling	27*27*96	-
8	C_2, 128 5x5x48 Conv, strd [1 1], pdng [2 2 2 2]	Grouped Conv	27*27*256	Wts 5*5*48*128, Bias 1*1*128*2
7	LKR_1, scale 0.01	Leaky ReLU	27*27*256	-
8	BN_2, 256 Chnl	Batch Norm	27*27*256	Offset 1*1*256, Scale 1*1*256
9	C_3 64 3x3x256 Conv, strd [11), pdng ’same’	Conv	27*27*64	Wts 3*3*256*64, Bias 1*1*64
10	LN_1, 64 Chnl	Layer Norm	27*27*64	Offset 1*1*64, Scale 1*1*64
11	Relu_2	ReLU	27*27*64	-
12	C_4 256 1x1x04 Conv, strd [1 1), pdng ’same’	Conv	27*27*256	Wts 1*1*64*256, Bias 1*1*256
13	BN_3, 256 Chnl	Batch Norm	27*27*256	Offset 1*1*256, Scale 1*1*256
14	C_5 64 1x1x256 Conv, strd [1 1), pdng ’same’	Conv	27*27*64	Wts 1*1*256*64, Bias 1*1*64
15	LKR_2, scale 0.01	Leaky ReLU	27*27*64	-
16	BN_4, 64 Chnl	Batch Norm	27*27*64	Offset 1*1*64, Scale 1*1*64
17	C_6 258 3*3*64 Conv, strd [1 1], pdng ’same’	Conv	27*27*256	Wts 3*3*64*256, Bias 1*1*256
18	BN_5, 256 Chnl	Batch Norm	27*27*256	Offset 1*1*256, Scale 1*1*256
19	Add_1 Element-wise Add, two inputs	Addition	27*27*256	-
20	C_7 258 3*3*258 Conv, strd [1 1), pdng ‘same’	Conv	27*27*256	Wts3*3*256*256, Bias 1*1*256
21	LKR_3, scale 0.01	Leaky ReLU	27*27*256	-
22	BN_6, 256 Chnl	Batch Norm	27*27*256	Offset 1*1*256, Scale 1*1*256
23	Pool2 3*3, strd [2 2], pdng [0 0 0 0]	Max Pooling	13*13*256	-
24	C_8 384 3*3*258 Conv, strd [1 1], pdng [1111]	Conv	13*13*384	Wts3*3*256*384, Bias 1*1*384
25	LKR_4, scale 0.01	Leaky ReLU	13*13*384	-
26	BN_7, 384 Chnl	Batch Norm	13*13*384	Offset 1*1*384, Scale 1*1*384
27	C_9 64 3*3*384 Conv, strd [1 1], pdng ’same’	Conv	13*13*64	Wts 3*3*384*64, Bias 1*1*64
28	LN_2, 64 Chnl	Layer Norm	13*13*64	Offset 1*1*64, Scale 1*1*64
29	Relu_3	ReLU	13*13*64	-
30	C_10 384 1*1*64 Conv, strd [1 1], pdng ’same’	Conv	13*13*384	Wts 1*1*64*384, Bias 1*1*384
31	BN_8, 384 Chnl	Batch Norm	13*13*384	Offset 1*1*384, Scale 1*1*384
32	C_11 64 1*1*384 Conv, strd [1 1], pdng ’same’	Conv	13*13*64	Wts 1*1*384*64, Bias 1*1*64
33	LKR _5, scale 0.01	Leaky ReLU	13*13*64	-
34	BN_9, 64 Chnl	Batch Norm	13*13*64	Offset 1*1*64, Scale 1*1*64
35	C_12 384 3*3*64 Conv, strd [1 1], pdng ’same’	Conv	13*13*384	Wts 3*3*64*384, Bias 1*1*384
36	BN_10, 384 Chnl	Batch Norm	13*13*384	Offset 1*1*384, Scale 1*1*384
37	Add_2 Element-wise Add, two inputs	Addition	13*13*384	-
38	C-13 2(192 3*3*192) Conv, strd [1 1], pdng [1111]	Grouped Conv	13*13*384	Wts 3*3*192*192, Bias 1*1*192*2
39	Relu_4	ReLU	13*13*384	-
40	BN_11, 384 Chnl	Batch Norm	13*13*384	Offset 1*1*384, Scale 1*1*384
41	C_14 384 5*5*384 Conv, strd [1 1],pdng ’same’	Conv	13*13*384	Wts 5*5*384*384, Bias 1*1*384
42	BN_12, 384 Chnl	Batch Norm	13*13*384	Offset 1*1*384, Scale 1*1*384
43	C_15 64 3*3*384 Conv, strd [1 1], pdng ’same’	Conv	13*13*64	Wts 3*3*384*64, Bias 1*1*64
44	C_16 64 1*1*384 Conv, strd [1 1], pdng ’same’	Conv	13*13*64 . .	Wts 1*1*384*64, Bias 1*1*64
45	LKR _6, scale 0.01	Leaky ReLU	13*13*64	-
46	LN_3, 64 Chnl	Layer Norm	13*13x64	Offset 1*1*64, Scale 1*1*64
47	Relu_5	ReLU	13*13*64	-
48	C_17 384 1*1x64 Conv, strd [1 1], pdng ’same’	Conv	13*13*384	Wts 1*1*64*384, Bias 1*1*384
49	BN_13, 384 Chnl	Batch Norm	13*13*384	Offset 1*1*384, Scale 1*1*384
50	BN_14 B, 64 Chnl	Batch Norm	13*13*64	Offset 1*1*64, Scale 1*1*64
51	C_18 384 3*3*64 Conv, strd [1 1], pdng ’same’	Conv	13*13*384	Wts 3*3*64*384, Bias 1*1*384
52	BN_15, 384 Chnl	Batch Norm	13*13*384	Offset 1*1*384, Scale 1*1*384
53	Add_3 Element-wise Add, two inputs	Addition	13*13*384	-
54	C-19 2 (128 3*3*192) Conv, strd [1 1], pdng [1111]	Grouped Conv	13*13*256	Wts 3*3*192*128, Bias 1*1*128*2
55	Relu_6	ReLU	13*13*256	-
56	BN_16, 256 Chnl	Batch Norm	13*13*256	Offset 1*1*256, Scale 1*1*256
57	Pool5 3*3, strd [2 2], pdng [0 0 0 0]	Max Pooling	6*6*256	-
58	FC_1 2048 layer	Fully Connected	1*1*2048	Wts 2048*9216, Bias 2048*1
59	Relu_7	ReLU	1*1*2048	-
60	Drop6 50% dropout	Dropout	1*1*2048	-
61	FC_2 2048 layer	Fully Connected	1x1x2048	Wts 2048x2048, Bias 2048x1
62	Relu-8	ReLU	1x1x2048	-
63	Drop7 50% dropout	Dropout	1x1x2048	-
64	FC_3 100 layer	Fully Connected	1x1x100	Wts 100x2048, Bias 100x1
65	Softmax	Softmax	1x1x100	-
66	Classoutput (’apple’ and 99 other classes)	Classification Output	-	-

The proposed framework starts from the data layer, accepting 227x227x3 images with the’ zerocenter’ Norm. Then C_1 96 11x11x3 (Conv) layer is used with strd [4 4) and pdng [0 0 0 0). (ReLU) Relu_l is followed by BN_1 (Batch Norm) layer with 96 Chnl and pool_1 3x3 (Max Pooling) with strd [2 2) and pdng [0 0 0 0]. Two (2) groups of C_2 of 128 5x5x48 (Conv) with strd [1 1] and pdng [2 2 2 2] is followed by LKR_1 (Leaky ReLU) with scale 0.01 and BN_2 (Batch Norm) with 256 Chnl. Batch Norm layer BN_2 is followed by the first branch of Conv layer C_3, 64 3x3x256 with strd [11) and pdng ’same’, LN_1 (Layer Norm) with 64 Chnl, Relu_2 (ReLU), C_4 256 1x1x04 (Conv) with strd [1 1) and pdng ’same’, BN_3 (Batch Norm) with 256 Chnl, C_5 64 1x1x256 (Conv) with strd [1 1) and pdng ’same’, LKR_2 (Leaky ReLU) with scale 0.01, BN_5 (Batch Norm) with 64 Chnl, C_6 258 3*3*64 (Conv) with strd [1 1] and pdng ’same’ and BN_4 (Batch Norm) with 256 Chnl. Then Add_1 (Addition layer) is used for element-wise addition of 2 inputs from (Batch Norm) layers BN_3 and BN_4. Add_1 is followed by Conv layer C_7 258 3*3*258 with strd [1 1) and pdng ‘same’, LKR_3 (Leaky ReLU) with scale 0.01, BN_6 (Batch Norm) with 256 Chnl, pool_2 3*3 (Max Pooling) with strd [2 2] and pdng [0 0 0 0], C_8 384 3*3*258 (Conv) with strd [1 1] and pdng [1111], LKR_4 (Leaky ReLU) with scale 0.01 and BN_7 (Batch Norm) with 384 Chnl. Batch Norm layer BN_7 is followed by the second branch of C_9 64 3*3*384 (Conv) with strd [1 1] and pdng ’same’, LN_2 (Layer Norm) with 64 Chnl, Relu_3 (ReLU), C_10 384 1*1*64 Conv with strd [1 1] and pdng ’same’, BN_8 (Batch Norm) with 384 Chnl, C_11 64 1*1*384 (Conv) with strd [1 1] and pdng ’same’, leaky Relu_5 (Leaky ReLU) with scale 0.01, BN_9 (Batch Norm) with 64 Chnl, C_12 384 3*3*64 (Conv) with strd [1 1] and pdng ’same’, BN_14 (Batch Norm) and BN_10 (Batch Norm) with 384 Chnl. Then, Add_2 (Addition layer) is used for the element-wise addition of two inputs from BN_8 and BN_10 (Batch Norm) layers. Add_1 is followed by two groups of Conv layers C_13 of 192 3*3*192 with strd [1 1] and pdng [1111], Relu_4 (ReLU), BN_11 (Batch Norm) with 384 Chnl, C_14 384 5*5*384 (Conv) with strd [1 1] and pdng ’same’ and BN_12 (Batch Norm) with 384 Chnl. (Batch Norm) BN_12 is followed by the third branch of C_15 64 3*3*384 (Conv) with strd [1 1] and pdng ’same’, C_16 64 1*1*384 (Conv) with strd [1 1] and pdng ’same’, LeakyRelu_6 (Leaky ReLU) with scale 0.01, LN_3Layer Norm with 64 Chnl, Relu_5 (ReLU), C_17 384 1*1x64 (Conv) with strd [1 1] and pdng ’same’, BN_13 (Batch Norm) with 384 Chnl, BN_14 (Batch Norm) with 64 Chnl, C_18 384 3*3*64 (Conv) with strd [1 1] and pdng ’same’ and BN_15 (Batch Norm) with 384 Chnl. Then, Add_3 (Addition layer) is used for the element-wise addition of two inputs from BN_13 and BN_15 (Batch Norm) layers. The Add_3 is followed by two groups of Conv layers C_19 128 3*3*192 with strd [1 1] and pdng [1111], Relu_6 (ReLU), BN_16 (Batch Norm) with 256 Chnl, pool_3 3*3 (Max Pooling) with strd [2 2] and pdng [0 0 0 0], FC_1 2048 (Fully Connected) layer, Relu_7 (ReLU), drop6 50% Dropout, FC_2 2048 Fully Connected layer, Relu_8 (ReLU), drop7 50% Dropout, FC_3 100 Fully Connected layer, softmax (Softmax) and Classoutput (crossentropyex) with output of classes classification.

In the proposed MBDLP-Net architecture, various common types of layers are utilized. The primary building blocks are convolutional layers, which apply filters to input images, resulting in activations. A feature map is created by repeatedly using the same filter for the input image. This feature map indicates the intensity and positions of recognized features in the input image. The innovation and novelty of convolutional neural networks (CNNs) lie in their ability to automatically learn numerous filters relevant to a training dataset, even for complex tasks like image classification. This allows for the detection of specific and essential features present anywhere on the input image. To generate a feature map, CNN uses a filter on[8 an input that summarizes the existence of observed features. For CNN, if there is an input size W x W x D and a Dout number of kernels with a spatial dimension of F, stride S, and padding value P, the size of the output produced may be calculated with the following equation:

$$W_{out}=\frac{W-F+2P}{S}+1$$ (6)

The convolutional layer can be expressed mathematically as:

$$h_{j}^{n}=\max(0,\sum_{k=1}^{K}h_{k}^{n-1}*w_{kj}^{n}$$ (7)

In Eq (7), *hjn is the output feature map*, *hkn*−1 *is the input feature map*, *and wkjn is the* kernel. ReLU is an activation function that stands for a rectified linear unit. It is mathematically defined as y = max (0, x). The most commonly used activation function in neural networks is ReLU, particularly in CNNs. If there is some uncertainty about what activation function to use while designing, a good first choice is usually ReLU. ReLU can be expressed mathematically as:

y=max(0,x)→∂f∂x=1ifx>=0else0y=max(0,x)→∂f∂x=1ifx>=0else0 (8)

Compared with tanh and sigmoid, Relu has a simpler function. Also, the gradient is constant, which does not require any computation in the backward direction (i.e., backpropagation). It is probably one of the first functions to have experimented with in network design.

The proposed strategy also uses some Batch Normalization layers. Batch_Norm [87] is an approach used for optimizing a channel of neurons throughout a short batch. It computes the mean and variance in segments. The standard deviation separates the features after computing the average/mean. The batch means *B* = *I*_1_…….*I*_*w*_ is expressed as under:

$${Mean}_{B}=\frac{1}{W}\;\sum_{z=1}^{w}I_{z}$$ (9)

In the scenario of Eq (9), the number of feature maps is denoted by w, and over a small batch, variance expression can be expressed as follows:

$${Var}_{B}=\frac{1}{W}\;\sum_{z=1}^{w}(I_{z-}{Mean}_{B}{)}^{2}$$ (10)

Therefore, further normalization is carried out by using an expression which is as follows:

Iz^=Iz−MeanBVarB+℧ (11)

Eq (11) ℧ is consistency, which is considered constant in this scenario. Both ReLU and Leaky ReLU processes are used in the proposed CNN design. The ReLU turns all numbers less than 0 to 0, which is written as under and in [88]:

$$I_{u,v}=max(\;0,I_{u,v})$$ (12)

For values lower than zero, leaky ReLU has a modest slope instead of zero. When you is negative, a leaky ReLU will have v = 0.01u. Further in-depth information about CNN can be learned from some other existing works [89–91].

**Fig 5:**
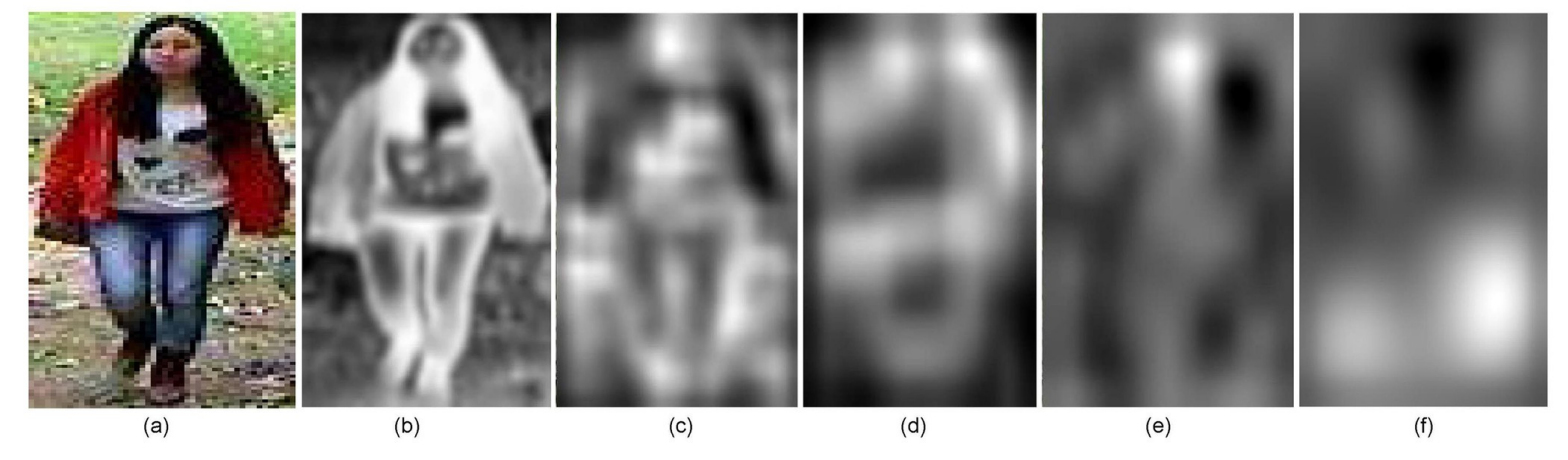
Visualizations at different convolutional layers (a) Conv1, (b) Conv2, (c) Convl3, (d) Conv4, and (e) Conv5 of the proposed MBDLP-Net of PKU-Reid dataset.

Image visualization at different convolutional layers Conv1, Conv2, Convl3, Conv4, and Conv5 of the proposed MBDLP-Net of the PKU-Reid dataset is presented in Fig 5.

### 3.3 Feature selection with ant colony optimization

For feature selection/optimization, ant colony selection (ACS) [92,93] is used, which is an approach based on learning. In ACS, the actions and activities of ants are mainly focused [94]. As they migrate from one location to another, the ants disseminate an ant deposit substance known as "pheromone." This material’s strength deteriorates with time. The ants follow the trail of pheromones. This helps to encourage ants to follow the less expensive route. That’s why, ants start to migrate from one position to another as vertex to vertex a network follows. A vertex represents a feature, and the ends connecting vertices provide the optimal selection to go on to the other next feature. The technique repeats until the best qualities are discovered. When the minimal number from vertices is reached, and the freezing condition is met, the technique grinds to a standstill. The vertices are connected in a mesh shape. Finally, the optimized feature set is fed into the classifiers for classification. At a given point in time, an ant’s feature selection is determined by the likelihood. Mathematically, the term of ACO is as follows:

Updating of pheromone

$$T_{xy}\leftarrow (1-\rho )T_{xy}+\sum_{k}\;\Delta \;{Tk}_{xy}$$ (13)

The left side of the equation indicates the amount of pheromone on the given edge x,y. The rate at which pheromones evaporate is represented by– *ρ*.

The amount of pheromone deposited is indicated by the final term on the right side.


$$\Delta \;{Tk}_{xy}={\{}_{0\;otherwise}^{Q/L_{k}\;if\;xy\;curve\;is\;used\;by\;ant\;k\;in\;its\;tour}$$ (14)

Q is a constant, and L is the cost of the length of an ant tour.

### 3.4 Classification

Classification is carried out by utilizing nine (9) different classifiers from which six (6) are SVM-based (Cubic (CSVM), coarse Gaussian (CGSVM), Median Gaussian (MGSVM), Fine Gaussian (FGSVM), Quadratic (QSVM) and Linear (LSVM)) and other three (3) are KNN based Cosine (CKNN), Coarse (RKNN), and Fine (FKNN). In the classification paradigm, the classifiers use 100, 250, 500, 750 & 1000 features set as input and predict the class label following feature computation to identify and categorize the poses of pedestrians based on eight (8) bins or angles. Several variables, including deep layer selection, activation function, and weight initialization influence the accuracy. The model’s accuracy is being improved via image preprocessing (dehazing). Classifiers are trained to anticipate the poses to get a high score accuracy.

## 4. Results and discussion

The primary and basic aim of the proposed work is to establish a deep learning-based CNN framework for dealing with the provided dataset. The MBDLP-Net CNN Network presented here only extracts most influential and powerful features after feature selection. Because this research aims to extract features on already existing datasets using the proposed CNN architecture, the pretraining is done on a third-party dataset, CIFAR-100. Furthermore, the proposed 66-layer MBDLP-Net CNN Network was developed after rigorous testing and assessment. Many different approaches are used to complete this architecture. The most common techniques involved were fine-tuning, layer addition, and removal. In its ultimate form, a 66-layer framework is proven to be good with the best performance results. This section explains the conversation and explanation of the proposed framework and its outcomes. This section starts by defining the dataset. Then, the process for performance evaluation is described. Lastly, the assessments and results are thoroughly described. For this manuscript, all the simulations and mentioned experiments are carried out on a Pentium Intel ® core i7-7700 @ 3.60 GHz. 16 GB RAM is present as system memory. The system is supported with GTX 1070 NVIDIA GeForce with GPU GP104 with 8GM dedicated memory with maximum digital resolution 7680x4320 @60 Hz. Windows 10 pro-64-bit (x64) is used as an Operating System. The coding and simulations are carried out using MATLAB R2021b and R2022b.

### 4.1 Datasets

This work employs three distinct datasets for performance evaluation. The first data set, PKU-Reid dataset, is used and is focused on pedestrians and has also been used for human re-identification. BDBO is the second dataset used for this work. The third dataset is TUD multiview pedestrians. Each dataset is comprised of 8 bin images with various 8 angled poses of different humans/pedestrians. Large datasets are necessarily required for ML processes to operate well during training. The dataset’s size is expanded by augmentation. Adding Gaussian noise, mirroring, color-shifting, and adding salt and pepper noise are the four major types of augmentations but in the proposed technique one kind of data augmentation (color-shifting) is utilized. The dataset became almost doubled in size. When a deep neural network is trained, the Data Augmentation techniques are supported in reducing overfitting. The majority of the enhancement processes described in image processing are used to improve image recognition or classification models.

To the best of our knowledge, pedestrian gender categorisation results were obtained utilising pioneering methodologies that were thoroughly investigated and tested on the PKU-Reid dataset. This dataset has served as a baseline for a variety of pedestrian-related studies since its inception in 2016. However, there is a significant lack in the literature on specialised studies on pedestrian pose estimation utilising the PKU-Reid dataset. This lack of precedence makes it difficult to directly compare the outcomes of our suggested strategy to existing methods, as there are no documented research or results in the literature that focus on this element.

Although the PKU-Reid dataset is a well-known resource for pedestrian re-identification and related tasks, no studies have used it particularly to assess a pedestrian’s posture. This lack of relevant literature emphasises the originality of our method while also highlighting the difficulty of confirming our findings against previous research. As a result, our findings must be understood with the awareness that they are an early investigation into this field, laying the groundwork for future research rather than giving a direct comparison with earlier techniques.

The BDBO is an essential resource that drives the advancement of sophisticated body orientation estimation techniques. Its extensive and meticulously annotated data allow researchers and practitioners to develop models that are highly accurate and applicable across various real-world scenarios. Characterized by a large volume of diverse samples, the BDBO is carefully curated to support research and development in body orientation analysis, providing the necessary data for training robust machine learning and computer vision models.

The details concerning to PKU-Reid, BDBO, and TUD Multiview Pedestrians datasets, including their specific characteristics, and how they contribute to the overall performance evaluation have also been provided.

Concerning to the probable limitations, and boundries, is is acknowledged that every dataset has its exclusive, and unique constraints that could affect the generalizability of results of proposed methodology. For example, because the PKU-Reid dataset is generally, and widely used for re-identification of pedestrians, it may not fully apprehend the range, and diversity of real-world situations. This could limit the generalization of the proposed model to unseen scenarios. Although, the BDBO dataset is effective for orientation estimation. It might have partialities due to the specific sides, angles, and poses it includes.

Another dataset, the TUD Multiview Pedestrians dataset, is recognized for its wide-ranging angles and multiview viewpoints. It proposes an appreciated diversity in pose estimation but there are also some challenges present. Because, it emphases on precise controlled situations, the dataset might not entirely imitate the complexities, and denesties of actual, and real-world pedestrian behavior. Furthermore, the 8-bin images from several angles may present certain partialities in pose estimation. Specifically when dealing with active, less common or more dynamic poses are not included in the dataset.

Data augmentation techniques are utilized to address these limitations, and to reduce overfitting. Specially, color-shifting augmentation technique is used, which effectively doubled the dataset size. It also has helped to enhance the model’s robustness. But, it is significant, and important to diagnose that the use of only one augmentation tehnique could still leave some characteristics under-explored. The detail of all three used datasets is elaborated in Table 2.

**Table 2:** No total augmented, and dehazed images are present in PKU-Reid, BDBO, and TUD datasets.

Sr No	Angle	No of images		Augmented images		Dehazed images		Normal Images	Augumented Images
		BDBO	PKU-Reid	BDBO	PKU-Reid	BDBO	PKU-Reid	TUD	TUD
1	0	3,981	228	7,962	456	7,962	456	546	1085
2	45	4,313	228	8,626	456	8,610	456	622	1240
3	90	4,172	228	8,344	452	8,312	452	400	798
4	135	4,639	228	9,278	448	9,230	448	749	1496
5	180	4,588	228	9,176	448	9,068	446	671	1340
6	225	4,640	228	9,280	448	9,112	444	722	1440
7	270	4,113	228	8,226	448	8,012	444	400	789
8	315	4,543	228	9,086	448	8,798	444	622	1242
**Total images**		**34,989**	**1,824**	**69,978**	**3,604**	**69,104**	**3,590**	**4,732**	**9,430**

### 4.2 Performance measures

The performance evaluation of the presented algorithms is taken place by using different formulas, some of which are Accuracy (ACC), TRP/Sensitivity (SE), TNR/Specificity (SP), Negative Prediction value (NPV), PRC/Positive Prediction (PPV), False Positive Rate (FPR), and False Negative Rate (FNR).

### 4.3 Experimental detail

To achieve the best outcomes, extensive testing of numerous iterations of particular feature sets is performed. In this section, a few important reviews are discussed. This section also explains a brief assessment of the accuracy of each carried out test. Numerous tests are carried out with varying quantities of feature sets during the feature selection process. These feature sets are used to automate the models used for prediction/ classification based on different classifiers of own choice. Overall CSVM has proved to be the best in all performance metrics in terms of ACC while Fine-KNN has the second-best overall performance in all performance metrics in terms of ACC.

#### 4.3.1 Experiment-I: Results on normal/hazy images of PKU-Reid dataset: Performance evaluation of normal/hazy images on PKU-Reid dataset by using five (5) features’ subsets (100, 250, 500, 750, and 1000 features) with SVM and KNN variants is expressed in Table 3

The best result such as 70.3% in terms of ACC is achieved by QSVM while using features subsets of 750 and 1000 features, while in terms of ACC as 69.69% is the second-best result which is obtained by CSVM with 750 features subset as shown in Table 3. The best results of 61.0%, 66.96%, 69.36%, 69.86%, and 70.3% ACC are attained using 100, 250, 500, 750, and 1000 features subsets, respectively with QSVM. It has been observed that QSVM performed better while predicting results under ACC protocol on normal/hazy images of the PKU-Reid dataset.

**Table 3:** Experimental results of normal/hazy images on PKU-Reid dataset by using five (5) features’ subsets (100, 250, 500, 750, and 1000 features) with SVM and KNN classifiers in terms of ACC.

Classifier	Features Selected Subsets					Measures					
	1	2	3	4	5	ACC	SE	SP	PR	FM	GM
CSVM	Π					0.61	0.61	0.96	0.67	0.94	0.39
		Π				0.67	0.67	0.96	0.73	0.95	0.33
			Π			0.69	0.70	0.97	0.75	0.96	0.31
				Π		**0.70**	0.68	0.97	0.76	0.95	0.30
					Π	0.69	0.68	0.96	0.72	0.95	0.31
CGSVM	Π					0.37	0.48	0.92	0.46	0.92	0.63
		Π				0.48	0.49	0.96	0.64	0.93	0.52
			Π			0.54	0.50	0.96	0.67	0.93	0.46
				Π		0.56	0.55	0.96	0.67	0.94	0.44
					Π	**0.58**	0.56	0.96	0.69	0.94	0.42
MGSVM	Π					0.54	0.54	0.94	0.57	0.93	0.46
		Π				0.61	0.64	0.96	0.68	0.95	0.39
			Π			**0.63**	0.63	0.96	0.71	0.95	0.36
				Π		0.62	0.62	0.95	0.63	0.94	0.38
					Π	0.59	0.64	0.95	0.63	0.95	0.40
FGSVM	Π					**0.52**	0.59	0.93	0.54	0.94	0.48
		Π				0.24	0.17	0.98	0.52	0.89	0.76
			Π			0.14	0.04	0.99	0.45	0.88	0.86
				Π		0.13	0.02	0.99	0.38	0.88	0.87
					Π	0.13	0.01	0.99	0.24	0.87	0.87
QSVM	Π					0.61	0.61	0.95	0.64	0.94	0.39
		Π				0.67	0.69	0.96	0.73	0.95	0.33
			Π			0.69	0.68	0.97	0.76	0.95	0.30
				Π		**0.70**	0.70	0.96	0.74	0.96	0.30
					Π	**0.70**	0.71	0.97	0.77	0.96	0.30
LSVM	Π					0.53	0.53	0.94	0.57	0.93	0.47
		Π				0.60	0.62	0.95	0.63	0.95	0.40
			Π			0.62	0.61	0.95	0.62	0.94	0.38
				Π		0.63	0.64	0.95	0.65	0.95	0.37
					Π	**0.64**	0.66	0.95	0.66	0.95	0.36
CKNN	Π					0.42	0.50	0.90	0.43	0.93	0.58
		Π				0.44	0.58	0.90	0.47	0.94	0.56
			Π			0.45	0.59	0.91	0.48	0.94	0.55
				Π		**0.46**	0.58	0.90	0.45	0.94	0.54
					Π	**0.46**	0.62	0.89	0.46	0.94	0.54
RKNN	Π					0.42	0.47	0.94	0.52	0.98	0.58
		Π				0.43	0.51	0.93	0.51	0.93	0.57
			Π			0.45	0.51	0.94	0.54	0.93	0.55
				Π		0.44	0.51	0.93	0.52	0.93	0.56
					Π	**0.46**	0.54	0.93	0.52	0.93	0.55
FKNN	Π					0.45	0.46	0.92	0.46	0.92	0.55
		Π				0.45	0.48	0.93	0.49	0.92	0.55
			Π			0.47	0.48	0.92	0.46	0.92	0.53
				Π		0.47	0.50	0.92	0.49	0.93	0.53
					Π	**0.48**	0.51	0.92	0.48	0.93	0.52

The results produced by each SVM and KNN variant in terms of ACC are given in Table 3. It is noted from the results that the best result such as 0.70% in terms of AUC is achieved by QSVM with both feature subsets of 750 and 1000 features. The second-best result of 0.70% in terms of AUC is produced by CSVM utilizing a 750 features subset. SVM variants performed better than KNN variants under ACC on normal/hazy images of the PKU-Reid dataset. A summary of the highest results in terms of ACC achieved by different classifiers on normal/hazy images of the PKU-Reid dataset is given in Fig 8. In terms of ACC, the best results on the PKU-Reid dataset are depicted in Table 4.

**Table 4:** Summary of experimental results of normal/hazy images on PKU-Reid dataset in terms of ACC by using five (5) features’ subsets (100, 250, 500, 750, and 1000 features) with SVM and KNN classifiers.

Classifier	Features selected					
	100	250	500	750	1000	ACC
CSVM				✓		**0.70**
CGSVM					✓	0.58
MGSVM			✓			0.63
FGSVM	✓					0.52
QSVM				✓	✓	**0.70**
LSVM					✓	0.64
CKNN				✓	✓	0.46
RKNN					✓	0.46
FKNN					✓	0.48

From the obtained results given in Table 4. the proposed technique has achieved 0.70, 0.58, 0.63, 0.52, 0.70, 0.64, 0.46, 0.46, and 0.48 results in terms of ACC utilizing CSVM, CGSVM, MGSVM, FGSVM, QSVM, and LSVM that are SVM based classifiers and CKNN, RKNN, and FKNN that are KNN based classifiers respectively on normal/hazy images of PKU-Reid dataset.

**Fig 6:**
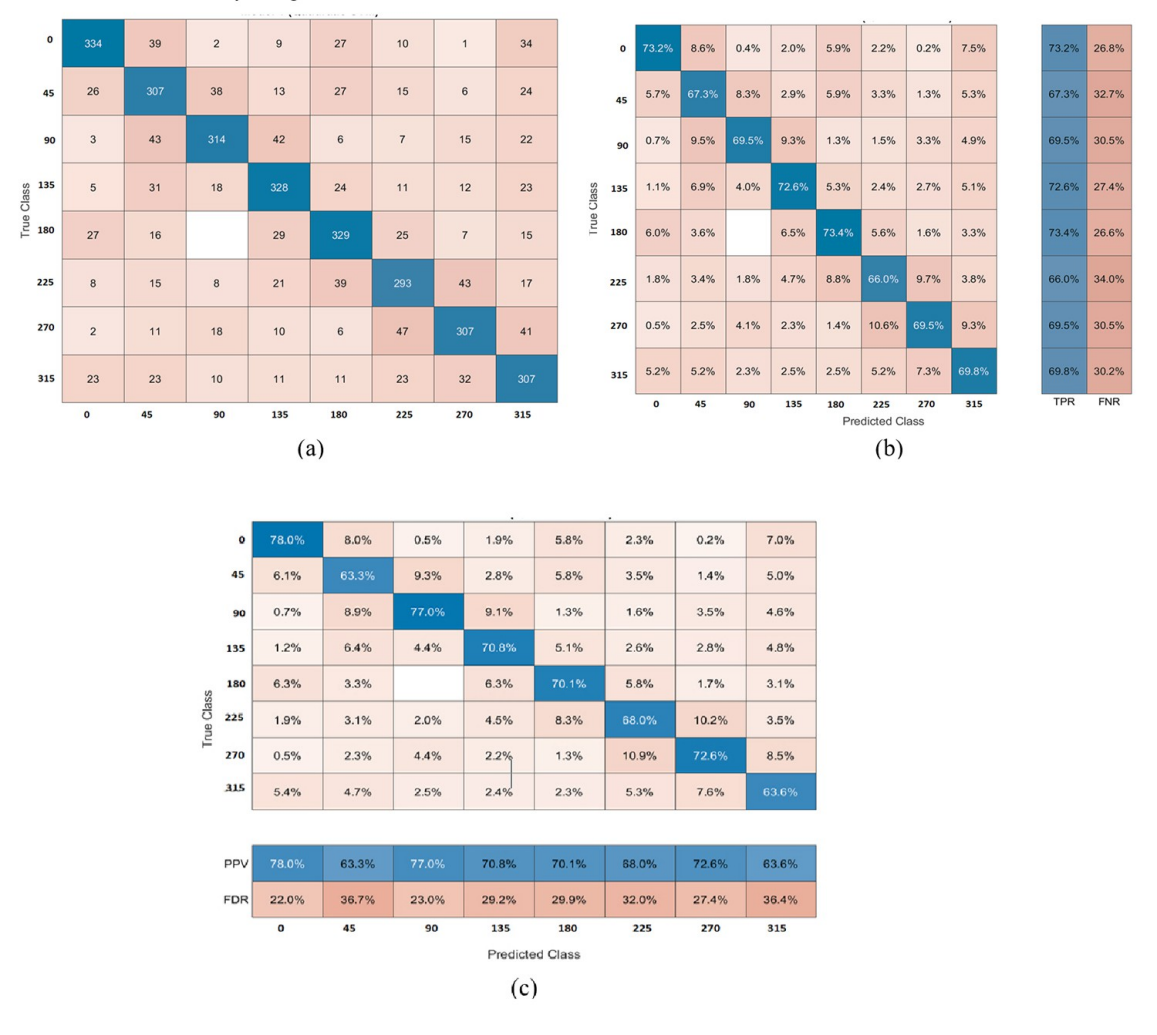
(a) The confusion matrix based on No of Observations, (b) The confusion matrix is based on TPR, and FNR, (c) The confusion matrix is based on PPV, and FDR on normal/hazy images of PKU-Reid dataset using Quadratic SVM.

**Fig 7:**
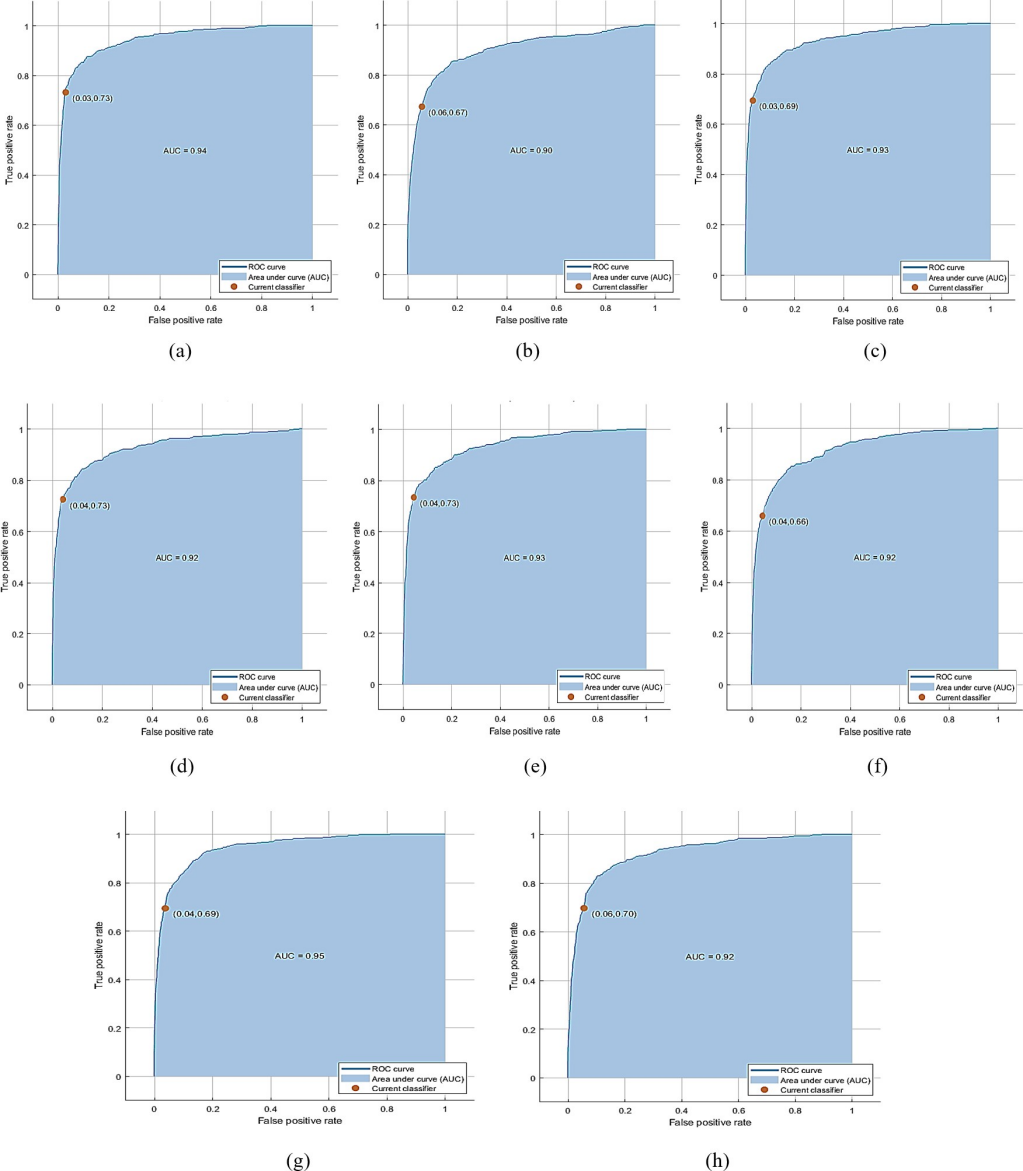
(a) 0, (b) 45, (c) 90, (d) 135, (e) 180, (f) 225, (g) 270, and (h) 315 class wise best ROC results on normal/hazy images of PKU-Reid dataset using Quadratic SVM.

The PKU-Reid dataset’s normal/hazy photos can be effectively processed using the proposed method to produce the best results. The best accuracy of 70.31% is achieved by QSVM with 1000 features subset and training time is 147.74 sec with prediction speed ~190 obs/sec. The total cost validation for this training is 1071. The confusion matrix based on No of Observations is given in Fig 6. (A), while based on TPR, FNR is shown in (b), and based on PPV, FDR is shown in (c) on normal/hazy images of PKU-Reid dataset. The class-wise best ROC results on normal/hazy images of the PKU-Reid dataset are expressed in Fig 7(A) 0, 7(b) 45, 7(c) 90,7(d) 135, 7(e) 180, 7(f) 225, 7(g) 270 and 7(h) 315 class wise best ROC results on normal/hazy images of PKU-Reid dataset.

#### 4.3.2 Experiment-II: Results on dehazed images of PKU-Reid dataset: Performance evaluation of dehazed images on PKU-Reid dataset by using five (5) features’ subsets (100, 250, 500, 750, and 1000 features) with SVM and KNN variants is expressed in Table 5

The best result such as 94.62% and 94.34% in terms of ACC is achieved by CSVM with features subsets of 750, and 1000 features respectively, whereas the second-best result such as 84.24%, 91.76%, 92.34%, 92.65%, and 93.23% in terms of ACC is obtained by QSVM with features subsets of 100, 250, 500, 750 and 1000 features respectively as shown in Table 5. The best results of 88.71%, 93.45%, 93.31%, 94.62%, and 94.34% ACC are attained using feature subsets of 100, 250, 500, 750, and 1000 features respectively with CSVM. It has been observed that CSVM performed better while predicting results under ACC protocol on dehazed images of the PKU-Reid dataset.

**Table 5:** Experimental results of dehazed images on PKU-Reid dataset by using five (5) features’ subsets (100, 250, 500, 750, and 1000 features) with SVM and KNNclassifiers in terms of ACC.

Classifier	Features Subsets					Measures					
	1	2	3	4	5	ACC	SE	SP	PR	FM	GM
CSVM	Π					0.89	0.90	0.99	0.91	0.99	0.11
		Π				**0.93**	0.96	0.99	0.94	0.99	0.07
			Π			**0.93**	0.93	0.99	0.94	0.99	0.07
				Π		**0.95**	0.96	0.99	0.96	0.99	0.05
					Π	**0.94**	0.96	0.99	0.95	0.99	0.06
CGSVM	Π					0.56	0.65	0.94	0.61	0.95	0.44
		Π				0.70	0.73	0.97	0.79	0.96	0.30
			Π			0.75	0.76	0.97	0.79	0.97	0.26
				Π		0.79	0.82	0.98	0.84	0.97	0.20
					Π	**0.81**	0.84	0.98	0.83	0.98	0.19
MGSVM	Π					0.74	0.79	0.97	0.77	0.97	0.20
		Π				0.86	0.89	0.98	0.89	0.98	0.14
			Π			**0.89**	0.90	0.99	0.90	0.99	0.11
				Π		**0.89**	0.92	0.99	0.91	0.99	0.11
					Π	0.88	0.91	0.98	0.89	0.99	0.12
FGSVM	Π					0.84	0.87	0.98	0.87	0.98	0.16
		Π				0.43	0.66	0.89	0.46	0.95	0.57
			Π			0.19	0.47	0.81	0.26	0.91	0.81
				Π		0.16	0.51	0.62	0.16	0.90	0.84
					Π	0.14	0.25	0.80	0.15	0.88	0.86
QSVM	Π					0.84	0.89	0.97	0.86	0.98	0.15
		Π				**0.92**	0.92	0.99	0.95	0.99	0.08
			Π			**0.92**	0.93	0.99	0.95	0.99	0.08
				Π		**0.93**	0.92	0.99	0.94	0.99	0.07
					Π	**0.93**	0.95	0.99	0.96	0.99	0.07
LSVM	Π					0.73	0.77	0.97	0.77	0.97	0.27
		Π				0.81	0.87	0.97	0.83	0.98	0.19
			Π			0.83	0.84	0.98	0.84	0.98	0.17
				Π		**0.85**	0.85	0.98	0.86	0.98	0.16
					Π	**0.85**	0.87	0.98	0.85	0.98	0.15
CKNN	Π					0.59	0.73	0.92	0.56	0.96	0.41
		Π				0.62	0.80	0.92	0.59	0.97	0.38
			Π			0.62	0.77	0.93	0.60	0.97	0.38
				Π		0.62	0.76	0.92	0.58	0.96	0.38
					Π	0.62	0.77	0.92	0.58	0.97	0.38
RKNN	Π					0.55	0.70	0.92	0.56	0.95	0.45
		Π				0.60	0.75	0.94	0.65	0.96	0.40
			Π			0.60	0.77	0.93	0.62	0.97	0.40
				Π		0.62	0.75	0.93	0.62	0.96	0.40
					Π	0.61	0.76	0.93	0.62	0.96	0.39
FKNN	Π					0.84	0.88	0.98	0.86	0.98	0.16
		Π				0.85	0.85	0.98	0.85	0.98	0.14
			Π			0.85	0.84	0.98	0.86	0.98	0.15
				Π		0.86	0.87	0.98	0.85	0.98	0.14
					Π	0.85	0.83	0.98	0.87	0.98	0.15

The results produced by each SVM and KNN variant in terms of ACC are given in Table 6. It is noted from the results that the best result such as 0.95 and 0.94 in terms of ACC is achieved by CSVM with both feature subsets 750 and 1000 features. The second-best result of 0.93% in terms of ACC is achieved by QSVM utilizing feature subsets of 750 and 1000 features. SVM variants performed better than KNN variants under ACC on dehazed images of the PKU-Reid dataset.

**Table 6:** Summary of experimental results of dehazed images on PKU-Reid dataset in terms of ACC by using five (5) features’ subsets (100, 250, 500, 750, and 1000 features) with SVM and KNN classifiers.

Classifier	Features					
	100	250	500	750	1000	ACC
CSVM				✓		**0.95**
CGSVM					✓	0.81
MGSVM			✓	✓		0.89
FGSVM	✓					0.84
QSVM				✓	✓	**0.93**
LSVM				✓	✓	0.85
CKNN		✓	✓	✓	✓	0.62
RKNN				✓		0.62
FKNN				✓		0.86

From Table 6, the proposed approach has achieved 0.95, 0.81, 0.89, 0.84, 0.93, 0.85, 0.62, 0.62, and 0.86 results in terms of ACC utilizing CSVM, CGSVM, MGSVM, FGSVM, QSVM, and LSVM that are SVM based classifiers and CKNN, RKNN, and FKNN that are KNN based classifiers respectively on dehazed images of PKU-Reid dataset.

**Fig 8:**
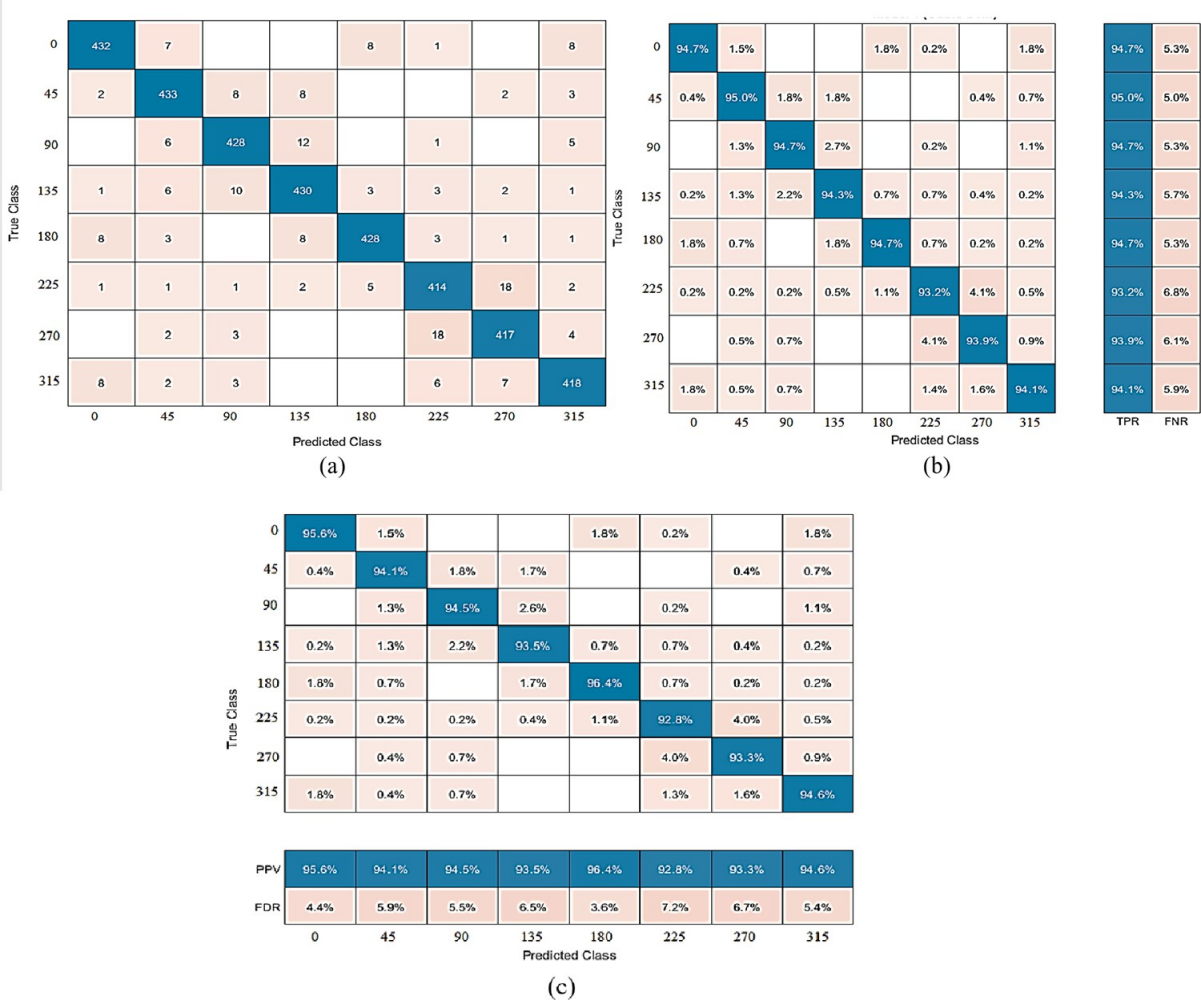
(a) The confusion matrix is based on No of Observations, (b) The confusion matrix is based on TPR, and FNR, and (c) The confusion matrix is based on PPV, and FDR on dehazed images of PKU-Reid dataset using Cubic SVM.

**Fig 9:**
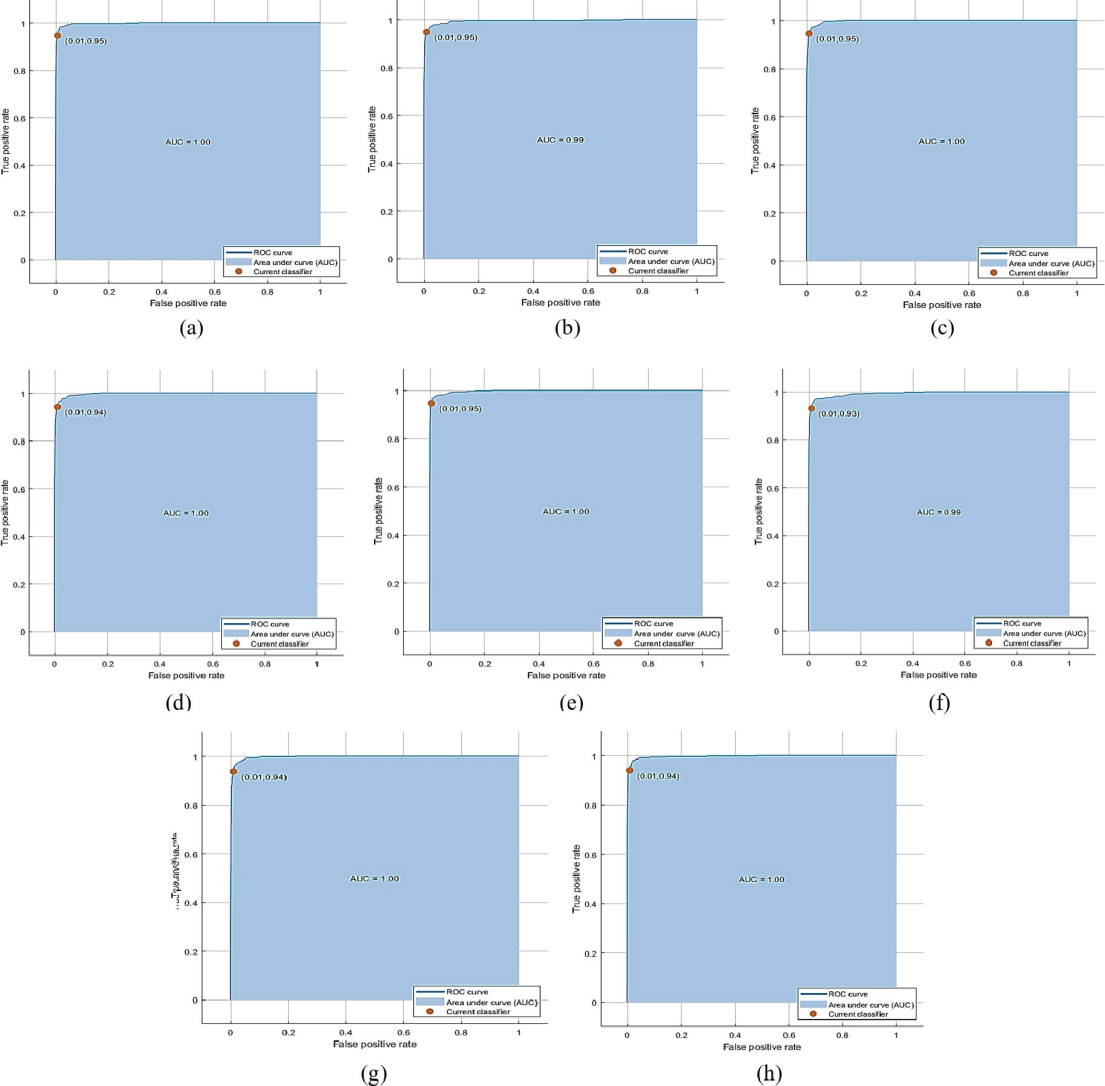
(a) 0, (b) 45, (c) 90, (d) 135, (e) 180, (f) 225, (g) 270, and (h) 315 class-wise best ROC results on dehazed images of PKU-Reid dataset using Cubic SVM.

The proposed method produces the best results on dehazed photos from the PKU-Reid dataset. The best accuracy of 94.62% is achieved by CSVM with 750 features subset and training time is 97.216 sec with prediction speed ~310 obs/sec. The total cost validation for this training is 204. The confusion matrix based on No of Observations is given in Fig 8(A), while based on TPR, FNR is shown in (b), and based on PPV, FDR is shown in (c) on dehazed images of PKU-Reid dataset. The class-wise best ROC results on dehazed images of the PKU-Reid dataset are expressed in Fig 9(A) 0, 9(B) 45, 9(C) 90, 9(D) 135, 9(E) 180, 9(F) 225, 9(G) 270 and 9(H) 315.

#### 4.3.3 Experiment-III: Results on standard/hazy images of BDBO dataset: Performance evaluation of standard/hazy images on BDBO dataset by using five (5) features’ subsets (100, 250, 500, 750, and 1000 features) with SVM and KNN variants is expressed in Table 7

The best result such as 92.8%, 93.0%, and 93.3% in terms of ACC is achieved by CSVM with features subsets of 500, 750, and 1000 features, whereas the second-best result, such as 91.9%, 91.9%, 91.9%, 92.0%, and 92.0% in terms of ACC is obtained by FKNN with features subset of 100, 250, 500, 750 and 1000 features as shown in Table 7. The best results of 92.8%, 93.0%, and 93.3% ACC are attained using feature subsets of 500, 750, and 1000 features, respectively with CSVM. It has been observed that CSVM performed better while predicting results under ACC protocol on standard/hazy images of the BDBO dataset.

**Table 7:** Experimental results of standard/hazy images on BDBO dataset by using five (5) features’ subsets (100, 250, 500, 750, and 1000 features) with SVM and KNN classifiers in terms of ACC.

Classifier	Features Subsets					Measures					
	1	2	3	4	5	ACC	SE	SP	PR	FM	GM
CSVM	Π					0.89	0.88	0.98	0.85	0.98	0.11
		Π				0.92	0.91	0.98	0.88	0.99	0.08
			Π			0.93	0.92	0.99	0.89	0.99	0.07
				Π		**0.93**	0.92	0.99	0.89	0.99	0.07
					Π	**0.93**	0.92	0.99	0.90	0.99	0.07
CGSVM	Π					0.50	0.62	0.94	0.59	0.95	0.50
		Π				0.62	0.75	0.95	0.69	0.97	0.38
			Π			0.70	0.81	0.96	0.74	0.97	0.30
				Π		0.75	0.83	0.97	0.77	0.98	0.25
					Π	0.79	0.85	0.97	0.78	0.98	0.21
MGSVM	Π					0.72	0.80	0.96	0.74	0.97	0.28
		Π				0.88	0.89	0.98	0.83	0.99	0.12
			Π			0.91	0.91	0.98	0.87	0.99	0.09
				Π		0.92	0.92	0.98	0.87	0.99	0.08
					Π	0.91	0.92	0.98	0.85	0.99	0.09
FGSVM	Π					0.90	0.91	0.97	0.82	0.99	0.10
		Π				0.72	0.56	1.00	0.97	0.95	0.28
			Π			0.36	0.08	1.00	0.91	0.89	0.64
				Π		0.23	0.07	1.00	0.92	0.89	0.77
					Π	0.18	0.06	1.00	0.93	0.89	0.82
QSVM	Π					0.82	0.83	0.97	0.79	0.98	0.20
		Π				0.87	0.88	0.98	0.84	0.98	0.13
			Π			0.89	0.89	0.98	0.85	0.99	0.11
				Π		0.90	0.89	0.98	0.85	0.99	0.10
					Π	0.91	0.90	0.98	0.86	0.99	0.10
LSVM	Π					0.53	0.68	0.95	0.62	0.96	0.47
		Π				0.66	0.77	0.96	0.72	0.97	0.34
			Π			0.71	0.80	0.97	0.76	0.97	0.29
				Π		0.73	0.81	0.97	0.76	0.98	0.27
					Π	0.71	0.82	0.97	0.77	0.98	0.26
CKNN	Π					0.84	0.85	0.97	0.78	0.98	0.16
		Π				0.85	0.87	0.97	0.78	0.98	0.15
			Π			0.86	0.89	0.97	0.79	0.98	0.14
				Π		0.86	0.88	0.97	0.79	0.98	0.14
					Π	0.86	0.88	0.97	0.79	0.98	0.14
RKNN	Π					0.70	0.79	0.95	0.68	0.97	0.30
		Π				0.73	0.81	0.95	0.67	0.97	0.28
			Π			0.73	0.83	0.95	0.68	0.98	0.27
				Π		0.73	0.83	0.95	0.68	0.99	0.27
					Π	0.74	0.83	0.95	0.68	0.98	0.26
FKNN	Π					**0.92**	0.93	0.99	0.92	0.99	0.08
		Π				**0.92**	0.93	0.99	0.92	0.99	0.08
			Π			**0.92**	0.93	0.99	0.92	0.99	0.08
				Π		**0.92**	0.94	0.99	0.93	0.99	0.08
					Π	**0.92**	0.94	0.99	0.93	0.99	0.08

The results produced by each SVM and KNN variant in terms of ACC are given in Table 8. It is noted from the results that the best result, 0.93% in terms of ACC, is achieved by CSVM with all feature subsets of 500, 750, and 1000 features. FKNN produces the second-best result of 0.92 in terms of ACC with all feature subsets of 100, 250, 500, 750, and 1000 features. SVM variants performed better than KNN variants under AUC on standard/hazy images of the BDBO dataset.

**Table 8:** Summary of experimental results of standard/hazy images on BDBO dataset in terms of ACC by using five (05) features’ subsets (100, 250, 500, 750, and 1000 features) with SVM and KNN classifiers.

Classifier	Features					
	100	250	500	750	1000	ACC
CSVM			✓	✓	✓	**0.93**
CGSVM					✓	0.79
MGSVM				✓		0.92
FGSVM	✓					0.90
QSVM					✓	0.91
LSVM				✓		0.73
CKNN			✓	✓	✓	0.86
RKNN					✓	0.74
FKNN	✓	✓	✓	✓	✓	**0.92**

From the results given in Table 8, the proposed approach has achieved 0.93, 0.79, 0.92, 0.90, 0.91, 0.73, 0.86, 0.74, and 0.92 results in terms of AUC utilizing CSVM, CGSVM, MGSVM, FGSVM, QSVM, and LSVM that are SVM based classifiers and CKNN, RKNN, and FKNN that are KNN based classifiers respectively, on standard/hazy images of BDBO dataset.

**Fig 10:**
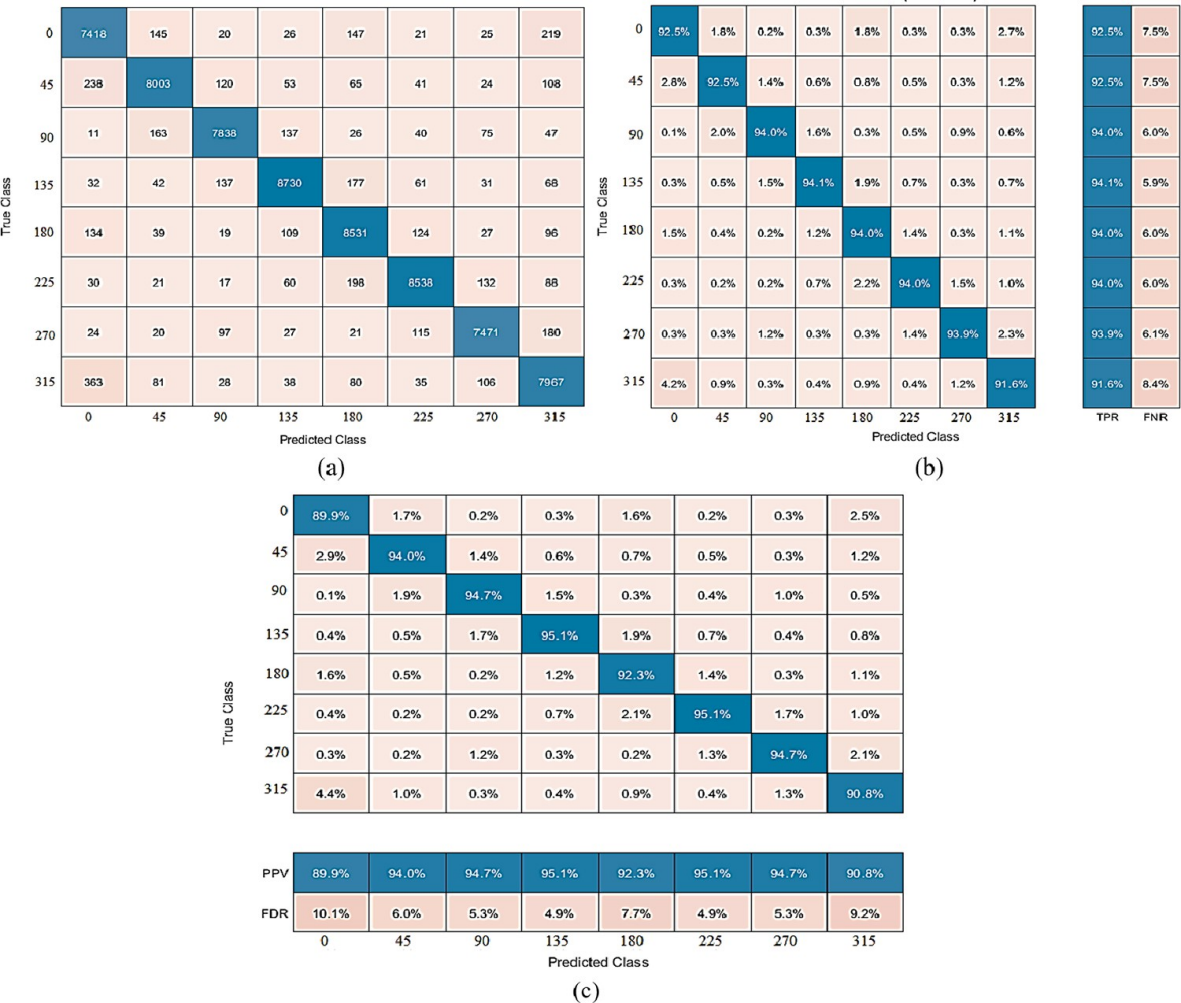
(a) The confusion matrix is based on No of Observations, (b) The confusion matrix is based on TPR and FNR, (c) The confusion matrix is based on PPV and FDR on normal/hazy images of the BDBO dataset using Cubic SVM.

**Fig 11:**
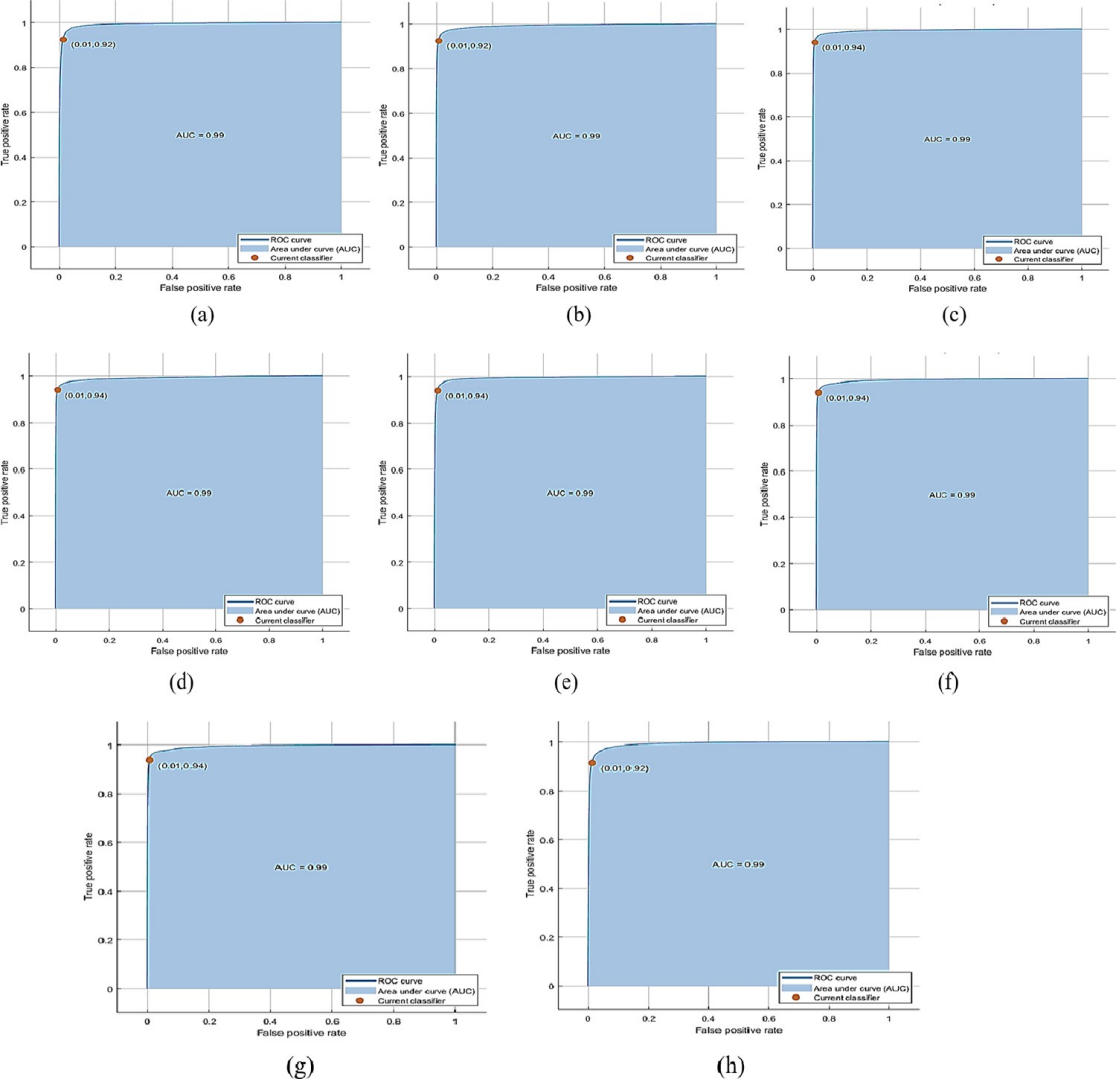
(a) 0, (b) 45, (c) 90, (d) 135, (e) 180, (f) 225, (g) 270, and (h) 315 class-wise best ROC results on standard/hazy images of BDBO dataset using Cubic SVM.

The proposed approach produces the best results on the BDBO dataset’s normal/hazy images. CSVM achieves the best accuracy of 93.30% with 1000 features subsets, and training time is 21069 sec with prediction speed ~21obs/sec. The total cost validation for this training is 4608. The confusion matrix based on The Observations is given in Fig 10(A), while based on TPR, FNR is shown in (b), and based on PPV, FDR is shown in (c) on normal/hazy images of the BDBO dataset. The class-wise best ROC results on standard/hazy images of the BDBO dataset are expressed in Fig 11(A) 0, 11(B) 45, 11(C) 90, 11(D) 135, 11(E) 180, 11(F) 225, 11(G) 270 and 11(H) 315.

#### 4.3.4 Experiment-IV: Results on dehazed images of BDBO dataset: Performance evaluation of dehazed images on BDBO dataset by using five (5) features’ subsets (100, 250, 500, 750, and 1000 features) with SVM and KNN variants is expressed in Table 9

The best result such as 94.7% and 94.8% in terms of ACC achieved by CSVM with features subsets of 750 and 1000 features, respectively, whereas the second-best result such as 93.7%, 94.2%, 94.1%, 93.9%, and 94.1% in terms of ACC is obtained by FKNN with features subsets of 100, 250, 500, 750, and 1000 features as shown in Table 9. The best results of 94.7% and 94.8% ACC are attained using 750 and 1000 feature subsets, respectively, with CSVM. It has been observed that CSVM performed better while predicting results under the ACC protocol on dehazed images on the BDBO dataset.

**Table 9:** Experimental results of dehazed images on BDBO dataset by using five (5) features’ subsets (100, 250, 500, 750, and 1000 features) with SVM and KNN classifiers in terms of ACC.

Classifier	Features Subsets					Measures					
	1	2	3	4	5	ACC	SE	SP	PR	FM	GM
CSVM	Π					0.91	0.90	0.98	0.87	0.99	0.09
		Π				**0.94**	0.92	0.99	0.90	0.99	0.06
			Π			**0.94**	0.93	0.99	0.91	0.99	0.06
				Π		**0.95**	0.94	0.99	0.92	0.99	0.05
					Π	**0.95**	0.94	0.99	0.91	0.99	0.05
CGSVM	Π					0.54	0.70	0.94	0.60	0.96	0.46
		Π				0.65	0.77	0.96	0.69	0.97	0.35
			Π			0.73	0.82	0.96	0.75	0.98	0.27
				Π		0.78	0.84	0.97	0.78	0.98	0.22
					Π	0.81	0.86	0.97	0.79	0.98	0.19
MGSVM	Π					0.75	0.83	0.96	0.75	0.98	0.25
		Π				0.89	0.90	0.98	0.84	0.99	0.11
			Π			**0.93**	0.93	0.98	0.89	0.99	0.07
				Π		**0.93**	0.93	0.98	0.88	0.99	0.07
					Π	**0.93**	0.93	0.98	0.87	0.99	0.07
FGSVM	Π					0.91	0.92	0.97	0.82	0.99	0.09
		Π				0.73	0.56	1.00	0.97	0.95	0.27
			Π			0.40	0.10	1.00	0.94	0.89	0.60
				Π		0.25	0.07	1.00	0.92	0.89	0.75
					Π	0.16	0.24	0.80	0.13	0.89	0.84
QSVM	Π					0.83	0.86	0.97	0.80	0.98	0.17
		Π				0.89	0.89	0.98	0.85	0.99	0.11
			Π			**0.91**	0.91	0.98	0.86	0.99	0.09
				Π		**0.91**	0.90	0.98	0.86	0.99	0.09
					Π	**0.91**	0.91	0.98	0.87	0.99	0.09
LSVM	Π					0.57	0.72	0.95	0.64	0.96	0.43
		Π				0.68	0.78	0.96	0.73	0.97	0.32
			Π			0.74	0.82	0.97	0.76	0.98	0.26
				Π		0.76	0.83	0.97	0.78	0.98	0.24
					Π	**0.78**	0.83	0.97	0.78	0.98	0.23
CKNN	Π					0.85	0.88	0.96	0.74	0.98	0.15
		Π				0.86	0.89	0.96	0.76	0.99	0.14
			Π			**0.87**	0.89	0.97	0.78	0.99	0.13
				Π		**0.87**	0.90	0.96	0.77	0.99	0.13
					Π	**0.87**	0.90	0.97	0.77	0.99	0.13
RKNN	Π					0.72	0.82	0.94	0.65	0.98	0.28
		Π				0.75	0.84	0.95	0.67	0.98	0.25
			Π			0.75	0.85	0.95	0.67	0.98	0.25
				Π		0.75	0.85	0.95	0.68	0.98	0.25
					Π	**0.76**	0.85	0.94	0.67	0.98	0.24
FKNN	Π					**0.94**	0.94	0.99	0.93	0.99	0.06
		Π				**0.94**	0.95	0.99	0.94	0.99	0.06
			Π			**0.94**	0.95	0.99	0.94	0.99	0.06
				Π		**0.94**	0.95	0.99	0.94	0.99	0.06
					Π	**0.94**	0.95	0.99	0.94	0.99	0.06

The results produced by each SVM and KNN variant in terms of ACC are given in Table 10. It is noted from the results that CSVM achieves the best result such as 0.95 in terms of ACC with both 750 and 1000 feature. The next-best result of 0.94 in terms of ACC is produced by FKNN utilizing feature subsets of 100, 250, 500, 750, and 1000 features. SVM variants performed better than KNN variants under ACC protocols on dehazed images of the BDBO dataset.

**Table 10:** Summary of experimental results of dehazed images on BDBO dataset in terms of ACC by using five (5) features’ subsets (100, 250, 500, 750, and 1000 features) with SVM and KNN classifiers.

Classifier	Features					
	100	250	500	750	1000	ACC
CSVM				✓	✓	**0.95**
CGSVM					✓	0.81
MGSVM			✓	✓	✓	0.93
FGSVM	✓					0.91
QSVM			✓	✓	✓	0.91
LSVM					✓	0.78
CKNN			✓	✓	✓	0.87
RKNN					✓	0.76
FKNN	✓	✓	✓	✓	✓	**0.94**

The results are given in Table 10. the proposed method has achieved 0.95, 0.81, 0.93, 0.91, 0.91, 0.78, 0.87, 0.76, and 0.94 results in terms of AUC utilizing CSVM, CGSVM, MGSVM, FGSVM, QSVM, and LSVM that are SVM based classifiers and CKNN, RKNN, and FKNN that are KNN based classifiers respectively, on dehazed images of BDBO dataset.

**Fig 12:**
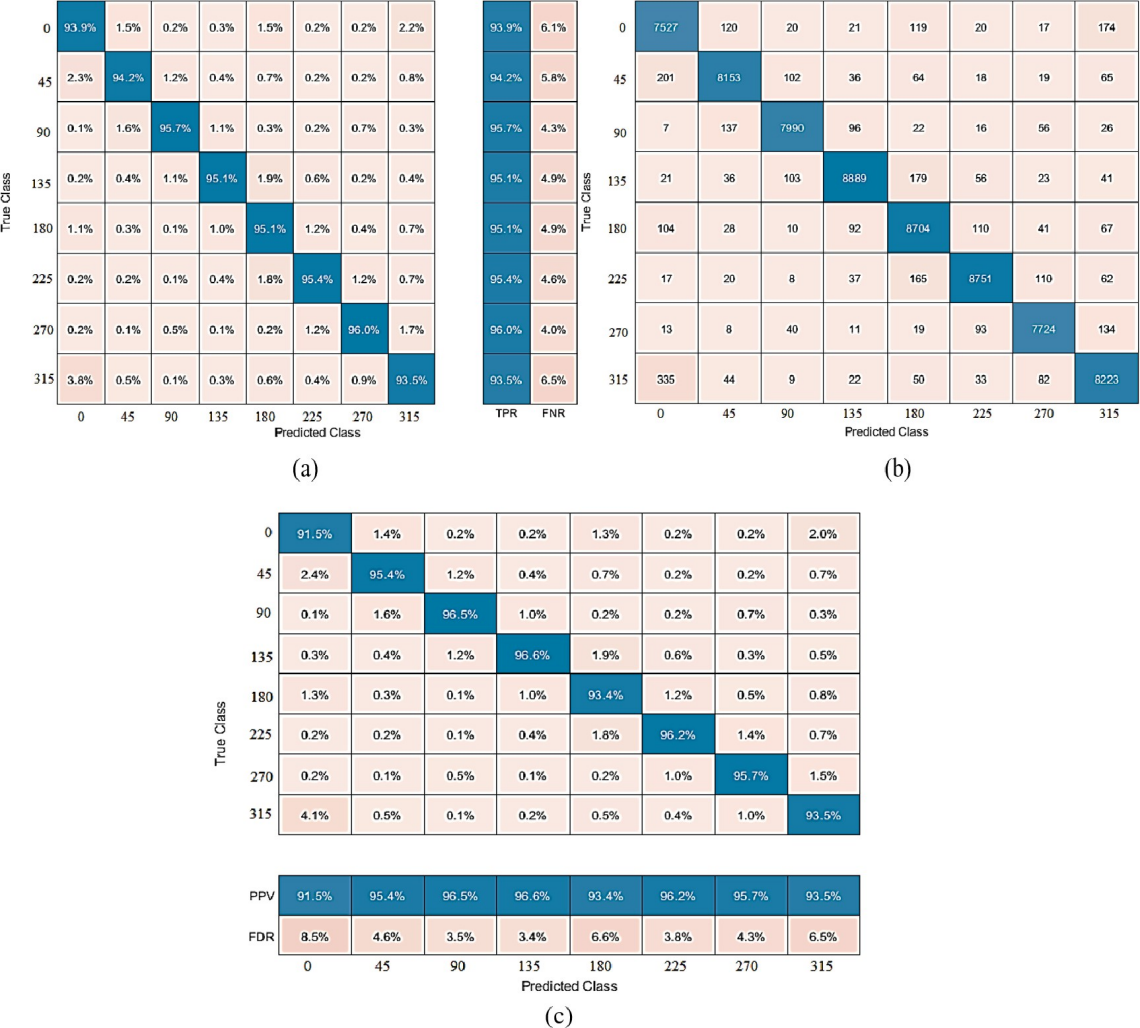
(a) The confusion matrix is based on No of Observations, (b) The confusion matrix is based on TPR and FNR, (c) The confusion matrix is based on PPV and FDR on dehazed images of the BDBO dataset using Cubic SVM.

**Fig 13:**
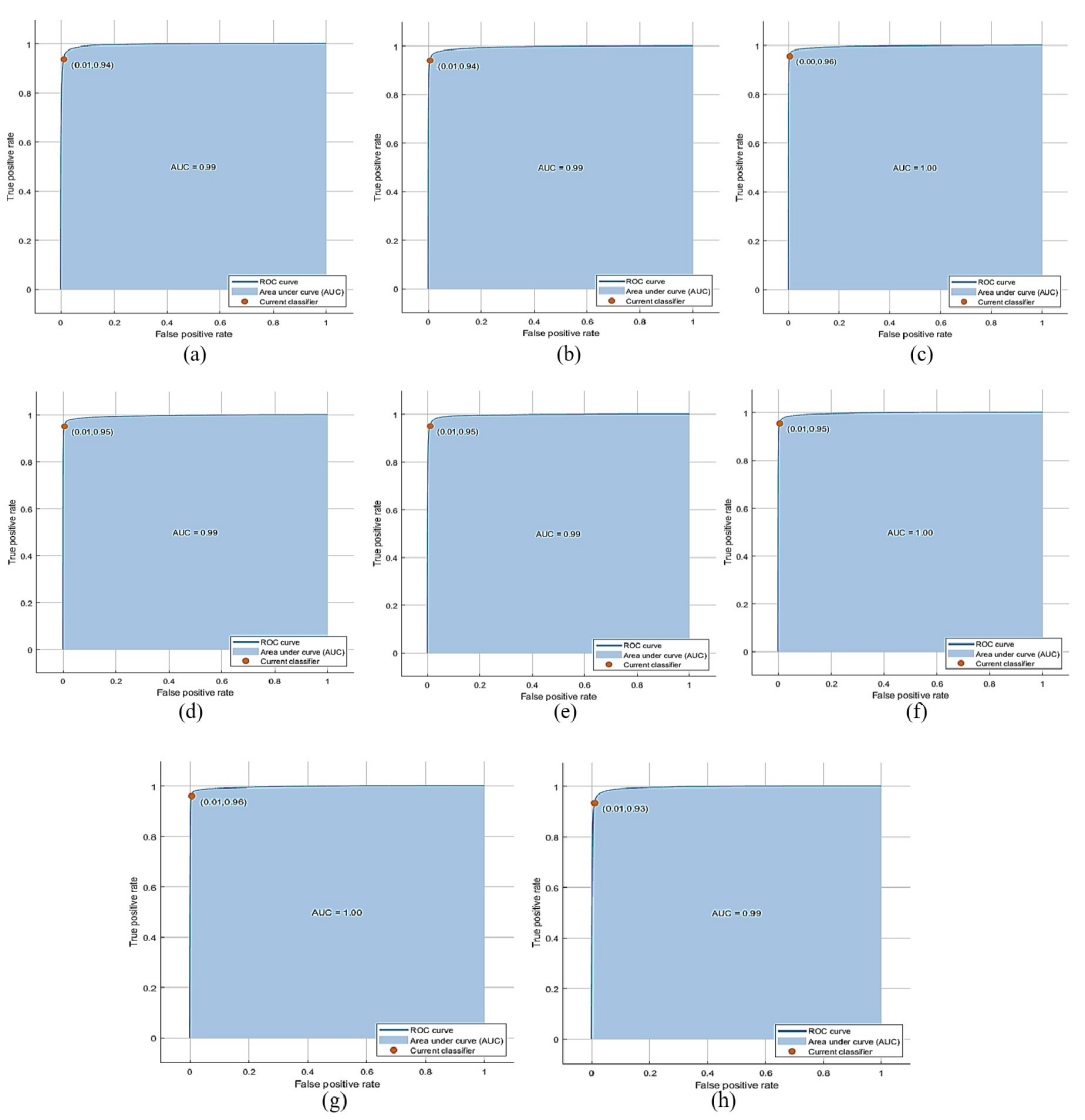
Class wise best ROC results on dehazed images of BDBO dataset using Cubic SVM (a) 0, (b) 45, (c) 90, (d) 135, (e) 180, (f) 225, (g) 270, and (h) 315.

The proposed approach performs well on dehazed photos from the BDBO dataset. CSVM achieves the best accuracy of 94.86% with 1000 features subset and training time is 19349 sec with prediction speed ~23obs/sec. The total cost validation for this training is 3579. The confusion matrix based on The Observations is given in Fig 12(A), while based on TPR, FNR is shown in (b), and based on PPV, FDR is shown in (c) on dehazed images of the BDBO dataset. The class-wise best ROC results on dehazed images of the BDBO dataset are expressed in Fig 13(A) 0, 13(B) 45, 13(C) 90, 13(D) 135, 13(E) 180, 13(F) 225, 13(G) 270 and 13(H) 315.

#### 4.3.5 Experiment-V: Results on dehazed images of BDBO dataset: Performance evaluation of dehazed images on BDBO dataset by using five (5) features’ subsets (100, 250, 500, 750, and 1000 features) using pretrained Inception Net with four (4) classifiers, i.e. CSVM, MGSVM, QSVM, and FKNN is expressed in Table 11

The results produced by each classifier, i.e., CSVM, MGSVM, QSVM, and FKNN, in terms of ACC, are given in Table 11. These results are obtained by using a pretrained Inception net. It is noted from the results that CSVM achieves the best result, such as 0.60 in terms of ACC with 750 features set, while the second best result is 0.58 with 500 features set. CSVM produces the next-best result of 0.56 in terms of ACC, and QSVM utilizing feature subsets of 250, and 750respectively.

**Table 11:** The results produced by each classifier, i.e. CSVM, MGSVM, QSVM, and FKNN in terms of ACC by using five (5) features’ subsets (100, 250, 500, 750, and 1000 features) using pretrained Inception Net.

CNN model	Classifier	Features Selected Subsets					Measures					
		1	2	3	4	5	ACC	SE	SP	PR	FM	GM
Inception net	CSVM	Π					0.49	0.83	0.97	0.80	0.98	0.51
			Π				0.56	0.88	0.98	0.88	0.98	0.44
				Π			0.58	0.89	0.99	0.89	0.99	0.42
					Π		**0.60**	0.91	0.99	0.90	0.99	0.40
						Π	0.48	0.73	0.99	0.90	0.99	0.52
	MGSVM	Π					0.43	0.77	0.95	0.69	0.97	0.57
			Π				0.52	0.86	0.98	0.82	0.98	0.48
				Π			0.53	0.89	0.98	0.88	0.99	0.47
					Π		0.51	0.89	0.99	0.89	0.99	0.49
						Π	0.50	0.88	0.98	0.88	0.98	0.47
	QSVM	Π					0.46	0.80	0.96	0.74	0.97	0.54
			Π				0.52	0.85	0.98	0.82	0.98	0.48
				Π			0.54	0.87	0.98	0.84	0.98	0.46
					Π		0.56	0.88	0.98	0.85	0.98	0.44
						Π	0.38	0.53	0.99	0.85	0.94	0.62
	FKNN	Π					0.42	0.92	0.99	0.92	0.99	0.58
			Π				0.41	0.93	0.99	0.93	0.99	0.59
				Π			0.41	0.94	0.99	0.93	0.99	0.58
					Π		0.41	0.94	0.99	0.93	0.99	0.59
						Π	0.40	0.92	0.99	0.92	0.99	0.58

#### 4.3.6 Experiment-VI: Experiment-V: Results on dehazed images of BDBO dataset: Performance evaluation of dehazed images on BDBO dataset by using five (5) features’ subsets (100, 250, 500, 750, and 1000 features) using pretrained VGG16 with four (4) classifiers i.e., CSVM, MGSVM, QSVM, and FKNN is expressed in Table 12

The results produced by each classifier, i.e., CSVM, MGSVM, QSVM, and FKNN, in terms of ACC, are given in Table 12. These results are obtained by using pretrained VGG16. It is noted from the results that CSVM achieves the best result, such as 0.80 in terms of ACC with both 750 and 1000 feature sets, while the second best result is 0.78 with 500 feature sets. The next-best result of 0.75 in terms of ACC is produced by QSVM utilizing features subset of 1000.

**Table 12:** The results produced by each classifier, i.e., CSVM, MGSVM, QSVM, and FKNN in terms of ACC by using five (5) features’ subsets (100, 250, 500, 750, and 1000 features using pretrained VGG16 Net.

CNN model	Classifier	Features Selected Subsets					Measures					
		1	2	3	4	5	ACC	SE	SP	PR	FM	GM
VGG16 net	CSVM	Π					0.66	0.87	0.98	0.84	0.98	0.34
			Π				0.74	0.89	0.98	0.87	0.99	0.26
				Π			0.78	0.91	0.99	0.89	0.99	0.22
					Π		**0.80**	0.91	0.98	0.89	0.99	0.21
						Π	**0.80**	0.92	0.99	0.89	0.99	0.20
	MGSVM	Π					0.51	0.80	0.96	0.74	0.97	0.49
			Π				0.66	0.87	0.98	0.83	0.98	0.34
				Π			0.71	0.91	0.98	0.88	0.99	0.29
					Π		0.70	0.91	0.99	0.89	0.99	0.30
						Π	0.60	0.91	0.98	0.89	0.99	0.29
	QSVM	Π					0.57	0.84	0.97	0.79	0/98	0.43
			Π				0.67	0.87	0.98	0.82	0.98	0.33
				Π			0.71	0.89	0.98	0.84	0.99	0.29
					Π		0.74	0.89	0.98	0.85	0.99	0.26
						Π	0.75	0.90	0.98	0.85	0.99	0.25
	FKNN	Π					0.50	0.91	0.99	0.91	0.99	0.50
			Π				0.50	0.91	0.99	0.91	0.99	0.50
				Π			0.50	0.92	0.99	0.91	0.99	0.50
					Π		0.50	0.92	0.99	0.92	0.99	0.50
						Π	0.50	0.91	0.99	0.91	0.99	0.50

#### 4.3.7 Experiment-VII: Results on dehazed images of BDBO dataset: Performance evaluation of dehazed images on BDBO dataset by using five (5) features’ subsets (100, 250, 500, 750, and 1000 features) using pretrained ResNet with four (4) classifiers, i.e. CSVM, MGSVM, QSVM, and FKNN is expressed in Table 13

The results produced by each classifier i.e. CSVM, MGSVM, QSVM, and FKNN in terms of ACC are given in Table 13. These results are obtained by using pretrained ResNet. It is noted from the results that CSVM achieves the best result, such as 0.69, in terms of ACC with both 1000 feature sets, while the second best result is 0.68 with 750 feature set. The next-best result of 0.66 in terms of ACC is also produced by CSVM utilizing features subset of 1000.

**Table 13:** The results produced by each classifier, i.e. CSVM, MGSVM, QSVM, and FKNN in terms of ACC by using five (5) features’ subsets (100, 250, 500, 750, and 1000 features) using pretrained ResNet.

CNN model	Classifier	Features Selected Subsets					Measures					
		1	2	3	4	5	ACC	SE	SP	PR	FM	GM
ResNet	CSVM	Π					0.55	0.83	0.98	0.83	0.98	0.45
			Π				0.63	0.89	0.98	0.88	0.99	0.37
				Π			0.66	0.91	0.99	0.90	0.99	0.34
					Π		0.68	0.91	0.99	0.90	0.99	0.32
						Π	**0.69**	0.92	0.99	0.91	0.99	0.30
	MGSVM	Π					0.46	0.77	0.96	0.71	0.97	0.54
			Π				0.57	0.88	0.98	0.83	0.98	0.43
				Π			0.59	0.91	0.98	0.88	0.99	0.42
					Π		0.57	0.92	0.99	0.90	0.99	0.43
						Π	0.25	0.37	0.97	0.66	0.92	0.75
	QSVM	Π					0.51	0.82	0.97	0.78	0.98	0.49
			Π				0.58	0.88	0.98	0.83	0.98	0.42
				Π			0.61	0.89	0.98	0.85	0.99	0.39
					Π		0.63	0.90	0.98	0.85	0.99	0.37
						Π	0.65	0.91	0.98	0.87	0.99	0.35
	FKNN	Π					0.44	0.89	0.99	0.90	0.99	0.56
			Π				0.43	0.91	0.99	0.92	0.99	0.57
				Π			0.42	0.92	0.99	0.92	0.99	0.58
					Π		0.42	0.92	0.99	0.92	0.99	0.58
						Π	0.41	0.92	0.99	0.92	0.99	0.58

#### 4.3.8 Experiment-VIII: Results on images of TUD dataset: Performance evaluation of images on TUD dataset by using five (5) features’ subsets (100, 250, 500, 750, and 1000 features) using MBDLP-Net CNN Network with four (4) classifiers i.e. CSVM, MGSVM, QSVM, and FKNN is expressed in Table 14

The results produced by each classifier, i.e., CSVM, MGSVM, QSVM, and FKNN, in terms of ACC, are given in Table 14. These results are obtained by using the MBDLP-Net CNN Network. It is noted from the results that CSVM achieves the best result such as 0.97 in terms of ACC with 100, 250, 500, 750, and 1000 features set while with MGSVM 0.97 is achieved with 1000 features set. With FKNN, 0.97 is achieved for 100, 250, 750, and 100 sets. The second best result is 0.96, which is achieved with QSVM with 100, 250, 500, 750, and 100 features set. MGSVM also achieves the second best result of 0.96 in terms of ACC with 500, and 750 features sets, and FKNN with 500 feature subset. A summary of experimental results of images on the TUD dataset in terms of ACC using five (5) features’ subsets (100, 250, 500, 750, and 1000 features) with variants of SVM and KNN classifiers is presented in Table 15.

**Table 14:** The results produced by each classifier, i.e., CSVM, MGSVM, QSVM, and FKNN, in terms of ACC by using five (5) features’ subsets (100, 250, 500, 750, and 1000 features) using MBDLP-Net CNN Network.

Classifier	Features Selected Subsets					Measures					
	1	2	3	4	5	ACC	SE	SP	PR	FM	GM
CSVM	Π					0.97	0.99	1.00	1.00	0.99	0.03
		Π				0.97	1.00	1.00	1.00	1.00	0.02
			Π			0.97	0.99	1.00	1.00	0.99	0.02
				Π		**0.97**	0.99	1.00	1.00	0.99	0.02
					Π	0.97	1.00	1.00	1.00	1.00	0.02
MGSVM	Π					0.90	0.99	1.00	0.99	0.99	0.10
		Π				0.94	1.00	1.00	1.00	1.00	0.05
			Π			0.96	0.99	1.00	1.00	0.99	0.03
				Π		0.96	0.99	1.00	1.00	0.99	0.03
					Π	0.97	0.99	1.00	1.00	0.99	0.02
QSVM	Π					0.96	0.99	1.00	1.00	0.99	0.03
		Π				0.96	1.00	1.00	1.00	1.00	0.03
			Π			0.96	0.99	1.00	1.00	0.99	0.03
				Π		0.96	0.99	1.00	1.00	0.99	0.03
					Π	0.96	1.00	1.00	1.00	1.00	0.03
FKNN	Π					0.97	0.99	1.00	1.00	0.99	0.03
		Π				0.97	1.00	1.00	1.00	1.00	0.02
			Π			0.96	0.99	1.00	1.00	0.99	0.03
				Π		0.97	1.00	1.00	1.00	1.00	0.02
					Π	0.97	0.99	1.00	1.00	0.99	0.02

**Table 15:** Summary of experimental results of images on TUD dataset in terms of ACC by using five (5) features’ subsets (100, 250, 500, 750, and 1000 features) with variants of SVM and KNN classifiers.

Classifier	Features					
	100	250	500	750	1000	ACC
CSVM	✓	✓	✓	✓	✓	**0.97**
MGSVM					✓	0.97
QSVM	✓	✓	✓	✓	✓	0.96
FKNN	✓	✓		✓	✓	**0.97**

## 5. Comparison with existing works

Two types of accuracy comparisons are carried out with various recently developed existing approaches in terms of obtained entire body orientation accuracies with the proposed method, and with pre-trained models or architectures.

The evaluated best performance of the proposed model using four (4) main classifiers, i.e., CSVM, MGSVM, QSVM, and FKNN, is compared with the results obtained from the same classifiers with pre-trained models or architectures such as Inception in Table 11, VGG16 in Table 12, and ResNet in Table 13. Comparison of existing pretrained and proposed systems in terms of the mean of complete body orientation accuracies Table 16 aims to demonstrate that the proposed network achieves higher accuracy and precision in estimating the poses of pedestrians. Specifically, the comparison highlights the superior performance of the proposed model in terms of its ability to accurately and precisely identify and estimate pedestrian poses, thereby validating its effectiveness over the traditional pretrained mentioned networks.

**Table 16:** Comparison of existing pretrained and proposed systems in terms of mean of complete body orientation accuracies.

Sr No	Dataset	Method	Accuracy %
1	**BDBO**	Layered CNN architecture	0.92
**2**		Inception	0.60
**3**		VGG16	0.80
**4**		ResNet	0.69
**5**		**Proposed** MBDLP-Net **system**	**0.95**

In comparison with recent existing approaches, the result of the proposed approach is found to be the best, robust, and superior. In terms of feature size, the proposed framework is examined for scalability concerns. It has been observed during experiments with increasing the feature size somewhat improves accuracy in results. The computation time increases as size of the features also increases. To address this, feature selection is used. Table 17 shows the direct comparison of existing approach with proposed system by using BDBO data set, and Table 18 shows the direct comparison of existing approach with proposed system by using TUD data set.

**Table 17:** Comparison of existing, and proposed system in terms of mean of full body orientation accuracies with BDBO dataset.

Sr No	Reference	Year	Dataset	Method	Accuracy %
1	[95]	2018	**BDBO**	Layered CNN architecture	0.92
**2**				**Proposed** MBDLP-Net **system**	**0.95**

**Table 18:** Comparison of existing, and proposed system in terms of mean of full body orientation accuracies with TUD dataset.

Sr No	Reference	Year	Dataset	Method	Accuracy %
1	[96]	2019	**TUD**	Teacher–student framework	0.786
2	[97]	2019		Two-stream network	0.890
3	[98]	2019		High-level semantic keypoints locator	0.809
4	[99]	2020		XGBoost	0.918
5	[100]	2021		CapsNet (Yolov3 algorithm)	0.930
6	[33]	2024		Transformer-based model architecture	0.785
**7**				**Proposed** MBDLP-Net **system**	**0.978**

The proposed method effectively addresses several limitations of existing techniques for pedestrian full-body pose and orientation estimation by introducing advanced, progressive approaches that result in improved performance. Traditional models often struggle with noise, particularly when working with color images, and may suffer from suboptimal feature selection. In contrast, our methodology utilizes grayscale images and incorporates dehazing as a preprocessing step, significantly enhancing visibility and accuracy. The 66-layer CNN model, featuring three distinct branches (B1, B2, B3), captures complex features of poses and orientations more effectively and is well-suited to handling still images with varying backgrounds. Additionally, ACS is used for feature optimization, ensuring that only the most relevant and appropriate features are used, thereby enhancing classification efficiency, robustness, and accuracy. The method has been tested across three independent datasets, consistently outperforming state-of-the-art models and achieving mean accuracies of 95% and 97%, demonstrating its efficiency, robustness, and effectiveness in various configurations.

## 6. Conclusion

Pose and orientation of a pedestrian in a specified direction, and his movement is occasionally correlated very loosely. As a conclusion, a deep CNN learning based method for recognizing pedestrian full-body pose/orientation MBDLP-Net is proposed. The proposed CNN approach tested individually for full body pose estimation on two publicly available datasets PKU-Reid and BDBO. This technique can be applied to every image sequence as well as to still images because full body appearance-based classification is used. After obtaining the results, comparison is carried out with stat-of-the-art existing techniques. The proposed method is found to be the best among other existing classification approaches with a prominent best outcome. For full-body pose estimation, **0.95%** value of accuracy is achieved with BDBO, and PKU-Reid while **0.97%** accuracy is achieved with TUD multiview pedestrians dataset. The accuracy achieved by proposed approach shows the robustness of the approach. For achieving more accurate results, the proposed CNN model may be fine-tuned further.

Furthermore, in this study, the proposed technique is applied to just eight orientation classes, but it may be extended to other orientations in the future for improved predictions. Furthermore, the proposed method can be beneficial for improved path prediction in people behavior analysis, danger assessment, people tracking, and in detecting human activities and actions.
