# Correction: Pedestrian POSE estimation using multi-branched deep learning pose net

**DOI:** 10.1371/journal.pone.0321410

**Published:** 2025-04-01

**Authors:** Muhammad Alyas Shahid, Mudassar Raza, Muhammad Sharif, Reem Alshenaifi, Seifedine Kadry

In the Funding statement, the project number from the funder Deanship of Postgraduate Studies and Scientific Research at Majmaah University is listed incorrectly. The correct project number is: R-2024-1336.
