# Expression of Concern: Pedestrian POSE estimation using multi-branched deep learning pose net

**DOI:** 10.1371/journal.pone.0361677

**Published:** 2026-10-29

**Authors:** The PLOS One Editors

Contrary to the Data Availability statement in [1,2], the individual-level underlying data supporting the article’s results were not provided with the article.

The corresponding author provided information on third-party datasets used in this article (provided here in S1 File). However, they did not provide the data underlying the figures and graphs on request, which the Editors consider to be necessary for compliance with the PLOS Data Availability policy. Therefore, the *PLOS One* Editors issue this Expression of Concern.

We regret that the issues were not addressed prior to the article’s publication.

Additionally, there are errors in the dataset count values in Table 2 of [1]. The corrected table is provided with this notice.

## Supporting information

S1 File Third-party datasets used in this study. (DOCX)

**Table 2:** No. of total normal, augmented, and dehazed images present in PKU-Reid, BDBO, and TUD datasets.

Sr No	Angle	No of images		Augmented images		Dehazed images		Normal Images	Augmented Images
**BDBO**	**PKU-Reid**	**BDBO**	**PKU-Reid**	**BDBO**	**PKU-Reid**	**TUD**	**TUD**
1	0	3,981	228	8,021	456	8,018	456	546	1,085
2	45	4,313	228	8,652	456	8,656	456	622	1,240
3	90	4,172	228	8,337	452	8,350	452	400	798
4	135	4,639	228	9,278	452	9,348	456	749	1,496
5	180	4,588	228	9,079	448	9,156	452	671	1,340
6	225	4,640	228	9,084	444	9,170	444	722	1,440
7	270	4,113	228	7,955	442	8,042	444	400	789
8	315	4,543	228	8,698	440	8,798	444	622	1,242
**Total images**		**34,989**	**1,824**	**69,104**	**3,590**	**69,538**	**3,604**	**4,732**	**9,430**

## References

[1] Shahid MA, Raza M, Sharif M, Alshenaifi R, Kadry S. Pedestrian POSE estimation using multi-branched deep learning pose net. PLoS One. 2025;20(1):e0312177. doi: 10.1371/journal.pone.0312177 39854382 PMC11761077

[2] Shahid MA, Raza M, Sharif M, Alshenaifi R, Kadry S. Pedestrian POSE estimation using multi-branched deep learning pose net. PLoS One. 2025;20(1):e0312177. doi: 10.1371/journal.pone.0312177 39854382 PMC11761077

