# Supplementary material for: Expression of Concern: Pedestrian POSE estimation using multi-branched deep learning pose net
Source: PLoS One. 2026 Oct 29;21(10):e0361677. doi: 10.1371/journal.pone.0361677 (PMC13718961; doi:10.1371/journal.pone.0361677)
Supplement: Supplementary file 1 [file pone.0361677.s001.docx]

**Pedestrian POSE estimation using multi-branched deep learning pose net**

Muhammad Alyas Shahid, Mudassar Raza, Muhammad Sharif, Reem Alshenaifi, Seifedine Kadry

**Data Availability:**

The CIFAR-100 dataset, used in this paper for training the proposed model, is a third-party dataset. The dataset is available at the following link: <https://www.cs.toronto.edu/~kriz/cifar.html>

**Apart, three more datasets are used in this research work: BDBO, PKU_REID, and TUD Multiview pedestrians.**

**BDBO:**

**BDBO** dataset is available at: <http://dx.doi.org/10.13140/RG.2.2.16841.56168>

**BDBO** Augmented dataset is available at**:** <https://doi.org/10.6084/m9.figshare.30576416.v3>

**BDBO Dehazed** dataset is available at**:** <https://doi.org/10.6084/m9.figshare.30576413.v3>

**PKU_REID:**

**PKU_REID** dataset is available at**:** <https://github.com/charliememory/PKU-Reid-Dataset>.

**PKU_REID Augmented** dataset is available at: <https://doi.org/10.6084/m9.figshare.30576416.v3>

**PKU_REID Dehazed** dataset is available at: <https://doi.org/10.6084/m9.figshare.30576413.v3>

In this research, the PKU_REID dataset is annotated and used for pose estimation. The dataset is therefore converted, and the mapping of the PKU_REID dataset images (image filename) with corresponding class labels (used in this work) is made available (in an Excel sheet) at:

<https://doi.org/10.6084/m9.figshare.30399904.v2>

**TUD Multiview pedestrian:**

**The TUD Multiview pedestrian’s** dataset is available at:

<https://www.mpi-inf.mpg.de/departments/computer-vision-and-machine-learning/research/people-detection-pose-estimation-and-tracking/monocular-3d-pose-estimation-and-tracking-by-detection>

**The TUD Multiview pedestrian’s Augmented** dataset is available at:

<https://doi.org/10.6084/m9.figshare.30576416.v3>

**The TUD Multiview pedestrian’s Dehazed** dataset is available at:

<https://doi.org/10.6084/m9.figshare.30576413.v3>

In this research, the TUD dataset is annotated, and the mappings of TUD dataset images (image filename) with corresponding class labels (used in this work) are made available (in an Excel sheet) at:

<https://doi.org/10.6084/m9.figshare.30399964.v1>
